# Comprehensive microRNA-seq transcriptomic profiling across 11 organs, 4 ages, and 2 sexes of Fischer 344 rats

**DOI:** 10.1038/s41597-022-01285-7

**Published:** 2022-05-12

**Authors:** Xintong Yao, Shanyue Sun, Yi Zi, Yaqing Liu, Jingcheng Yang, Luyao Ren, Guangchun Chen, Zehui Cao, Wanwan Hou, Yueqiang Song, Jun Shang, He Jiang, Zhihui Li, Haiyan Wang, Peipei Zhang, Leming Shi, Quan-Zhen Li, Ying Yu, Yuanting Zheng

**Affiliations:** 1 State Key Laboratory of Genetic Engineering, Human Phenome Institute, School of Life Sciences and Shanghai Cancer Center, Fudan University, Shanghai, 200438 China; 2 Department of Immunology, Microarray and Immune Phenotyping Core Facility, University of Texas Southwestern Medical Center, Dallas, TX 75390 USA

**Keywords:** Non-coding RNAs, Transcriptomics

## Abstract

Rat is one of the most widely-used models in chemical safety evaluation and biomedical research. However, the knowledge about its microRNA (miRNA) expression patterns across multiple organs and various developmental stages is still limited. Here, we constructed a comprehensive rat miRNA expression BodyMap using a diverse collection of 320 RNA samples from 11 organs of both sexes of juvenile, adolescent, adult and aged Fischer 344 rats with four biological replicates per group. Following the Illumina TruSeq Small RNA protocol, an average of 5.1 million 50 bp single-end reads was generated per sample, yielding a total of 1.6 billion reads. The quality of the resulting miRNA-seq data was deemed to be high from raw sequences, mapped sequences, and biological reproducibility. Importantly, aliquots of the same RNA samples have previously been used to construct the mRNA BodyMap. The currently presented miRNA-seq dataset along with the existing mRNA-seq dataset from the same RNA samples provides a unique resource for studying the expression characteristics of existing and novel miRNAs, and for integrative analysis of miRNA-mRNA interactions, thereby facilitating better utilization of rats for biomarker discovery.

## Abstract (summary)

**Table:** 

Measurement(s)	miRNA expression profiling
Technology Type(s)	Next Generation Sequencing
Factor Type(s)	organ • age • sex
Sample Characteristic - Organism	Rattus norvegicus

## Background & Summary

MicroRNAs (miRNAs) are small noncoding RNAs (21–24 nucleotides) that mediate post-transcriptional regulation of gene expression by binding to the 3′ untranslated regions of messenger RNAs (mRNA)^1–5^. miRNAs play pivotal roles in biological processes such as differentiation, development, tissue growth, and tumor initiation and progression^2,4,6^. miRNAs can serve as potential clinical biomarkers for disease diagnosis, prognosis, and treatment selection^7–9^, as well as therapeutic targets for human diseases^10–12^. However, the development of miRNA-based biomarkers and therapies is hampered due to incomplete understanding of the characteristics of miRNA profiles across different organs, various developmental stages, or sexes of rat, an extensively used animal model for evaluating drug safety and understanding drug mechanisms of action. Furthermore, the rat miRNA transcriptome is much less well annotated compared to that of mouse and human, with 764, 1915, and 2588 miRNAs annotated in miRBase v.22.1 (http://www.mirbase.org/) for rat, mouse, and human, respectively^13,14^. Therefore, characterizing rat miRNA expression profiles more comprehensively is warranted, especially with the advancement of high-throughput technologies such as microarrays and next-generation sequencing.

Several efforts for constructing a catalog of rat miRNA expression have been reported. For example, Minami *et al.*^15^ reported a dataset of microarray-based expression profiles of 424 miRNAs cross 55 different organs and tissues of 10-week-old male Sprague-Dawley rats. Similarly, Bushel *et al.*^16^ developed the RATEmiRs database consisting of miRNA-seq expression data across 14 organs among 12/13-week-old Sprague Dawley rats of both sexes^17^, through six sequencing batches. These studies mainly focused on miRNA expression differences among various organs. However, the diversity and number of expressed miRNAs in animals is not only organ-specific^18,19^, but also shows age dependence^1,20,21^ and sex biases^22,23^. Specifically, the role of miRNAs in the aging process has often been overlooked and is therefore largely unclear.

Ideally, studies on rat transcriptome should simultaneously cover multiple organs across different developmental stages for both sexes. Indeed, in a widely cited previous study^24,25^ as part of the Sequencing Quality Control (SEQC) consortium^26^, we constructed a comprehensive rat transcriptomic BodyMap with RNA-seq of 320 samples from 11 organs of both sexes of juvenile, adolescence, adult, and aged Fischer 344 rats and identified a large number of transcripts showing organ-specific, age-dependent, or sex-specific expression patterns. The unique rat mRNA dataset has already been widely used by scientific communities to advance our understanding of the dynamics of rat transcriptome, including studies on circRNAs^27–29^, the development of annotation tools^30,31^, and enrichment of database resources^32,33^.

**Fig. 1:**
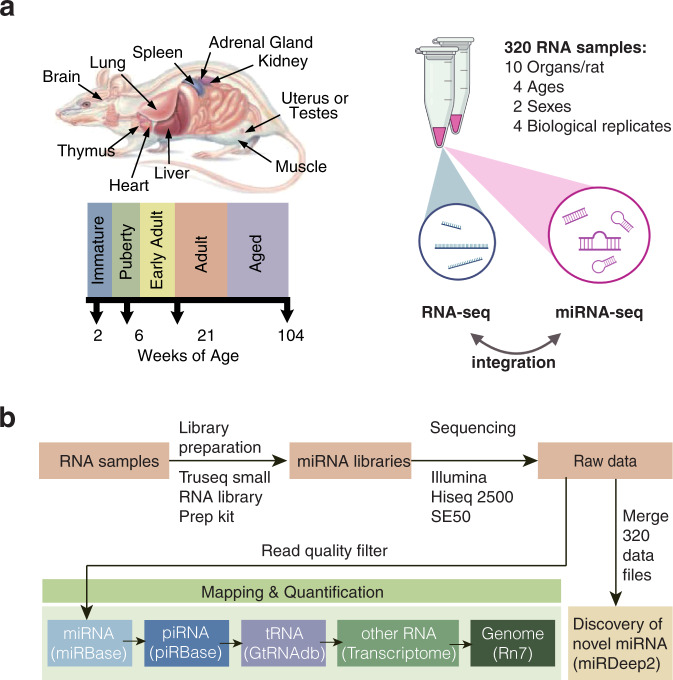
Overview of study design. 320 total RNA samples were collected from 16 female rats and 16 male rats of the Fisher 344 strain, including four rats for each sex under each of the four developmental stages, and ten organs (adrenal gland, brain, heart, kidney, liver, lung, muscle, spleen, thymus, testis, or uterus) per rat. Aliquots of the same RNA samples have also been used to construct mRNA dataset previously^24,25^ (**a**), which makes it possible to integrate miRNA data with mRNA data. (**b**) Schematic overview of the miRNA-seq workflow: miRNA libraries were prepared and sequenced for each of the 320 samples. For miRNA quantification, adapter sequences were removed, followed by a read-quality filter. Reads of high quality were then mapped to miRBase, piRBase, GtRNAdb, and the NCBI rat transcriptome and genome. In addition, reads from all 320 samples were pooled for novel miRNA discovery.

Here, as a follow-up and complementary study, we constructed a comprehensive rat miRNA BodyMap by generating miRNA-seq data from the same 320 samples and described the quality and the dynamic characteristics of miRNA expression profiles across 11 organs, between two sexes, and over the life span of the rats (Fig. 1). We quantified 604 out of the 764 miRNAs annotated in miRbase v22.1 (http://www.mirbase.org/). In addition, to explore the potential of novel miRNA discovery with this dataset, we used the miRDeep2 algorithm^34,35^ and identified 12 organ-enriched novel miRNA candidates. The miRNA-seq expression data were then analyzed in conjunction with the corresponding mRNA expression profiles. The quality of the miRNA-seq data was found to be high as seen from various aspects such as raw sequences, mapped sequences, and biological replicates.

The dataset we present here can help researchers study the characteristics of rat transcriptome and identify novel miRNAs. In addition, along with the mRNA dataset from the same set of samples, this miRNA-seq dataset provides a unique resource for data integration.

## Methods

### Organ collection and RNA isolation

Female and male Fischer 344 rats (pair-housed under standard conditions) from the National Center for Toxicological Research of the US Food and Drug Administration animal-breeding colony were euthanized by carbon dioxide asphyxiation at 2, 6, 21 and 104 week-of-age as previously described^24,25^. In summary, 320 tissue samples were derived from 16 female and 16 male rats of the Fischer 344 strain, including four rats for each sex under each of the four developmental stages and ten organs (adrenal gland, brain, heart, kidney, liver, lung, muscle, spleen, thymus, and testis or uterus) per rat. That is, four biological replicates per sample group were used in this study. Ground organ tissue was stored at −80 °C. Total RNA was extracted from ground tissue by using the miRNeasy Mini Kit (Qiagen) according to the manufacturer’s protocol, including treatment with DNase. RNAs longer than 18 nucleotides were recovered with this method. The quality of each RNA sample was examined with an Agilent 2100 Bioanalyzer.

### Construction and sequencing of miRNA-seq libraries

Small RNA libraries were constructed using Illumina TruSeq Small RNA Library Prep kit (the sequences of adapters and primers are provided in Table 1) by following the manufacturer’s Reference Guide with some modifications. In brief, 1 µg of total RNA (including miRNA) as input was sequentially ligated with 3′ and 5′ adapters, and the ligated RNA fragments were then reverse transcribed to single-stranded cDNAs using Superscript II reverse transcriptase (Invitrogen) and RT primer. The cDNAs were amplified by PCR with 13 cycles, and the resulting products were further purified with 2× Agencourt AMPure XP beads (Beckman), followed by size selection using the Pippin Prep Size Selection System (Sage Science). The final libraries were validated using Agilent High Sensitivity DNA assay, and subjected to 50 bp single-end sequencing (SE50) on an Illumina HiSeq. 2500. No spike-in controls or negative controls were used in the construction of this dataset.

**Table 1:** Sequence information of the Truseq-small RNA kit.

Name	Sequence
3′adapter	5′TGGAATTCTCGGGTGCCAAGG
5′adapter	5′GUUCAGAGUUCUACAGUCCGACGAUC
PCR primer (RP1)	5'AATGATACGGCGACCACCGAGATCTACACGTTCAGAGTTCTACAGTCCGA
PCR index primer	5′ CAAGCAGAAGACGGCATACGAGAT[6 bases]GTGACTGGAGTTCCTTGGCACCCGAGAATTCCA

### Pre-processing and processing of the reads

The adapter sequences were removed with fastp 0.23.2 software (http://opengene.org/fastp/fastp.0.23.2), followed by a quality filter and a length filter. Reads with more than 2 “N” bases were discarded. The clipped reads with length between 16 and 35 nt were retained for alignment to various transcript types, including miRNA (miRbase v22.1, http://www.mirbase.org/), piRNA (piRBase, http://www.regulatoryrna.org/database/piRNA/download.html), tRNA (GtRNAdb, http://gtrnadb.ucsc.edu/GtRNAdb2/genomes/eukaryota/Rnorv7/Rnorv7-seq.html), other RNA (NCBI transcriptome, https://www.ncbi.nlm.nih.gov/genome/73), and the rat genome (Rn7, https://hgdownload.soe.ucsc.edu/downloads.html#mammals). The alignment was performed with Bowtie 1.3.1 (http://bowtie-bio.sourceforge.net/manual.shtml) with less than 2 mismatched bases allowed.

### Filtering of miRNAs and samples

The following strategies were used to filter miRNAs and samples of questionable quality: (1) the low-expressed miRNAs less than one count per library on average, were discarded. (2) a sample was removed if it failed to cluster with other samples from the same organ type in a hierarchical clustering analysis. Spl_F_104_4 and Brn_M_006_3 samples were removed, as they clustered to uterus samples instead of spleen and brain, respectively. Ultimately, a miRNA expression matrix with 604 miRNAs and 318 samples was obtained for further analysis.

### Identification of novel miRNA candidates

First, reads from all 320 samples were pooled for identification of novel miRNA candidates. Using the mapper module provided within miRDeep2.0.1.3, raw reads of the merged samples were subjected to a series of stringent filters (discarding low-quality reads and reads with fewer than 18 nt after clipping the 3′ adapter), and the remaining sequences were then mapped to the rat genome reference (Rn7). Next, the mapped reads were submitted to the miRDeep2 module to detect novel miRNAs with default parameters. Novel miRNAs that have passed the stringent filters (miRDeep2 score ≥10, significant Ranfold *P*-value and no rfam alert of the possibility of being a rRNA) were selected as the potential novel miRNA candidates.

To validate the organ-specificity of the novel miRNA candidates, we quantified the novel miRNA expressions across 320 samples and performed differential expression analysis between any two organs at each developmental stage. A miRNA is considered ‘organ-specific’ if it is over-expressed by at least 1.5 fold (fold-change > 1.5 and adjusted *P*-value < 0.05) in one organ over all other organs and across all four developmental stages. Finally, the 12 organ-specific sequences were reported as novel miRNA candidates in Online-only Table 1.

## Data Records

The miRNA-seq dataset generated in study is available in the NCBI Gene Expression Omnibus (GEO) with series accession number GSE172269^36^. This accession contains both the raw sequence data files (fq.gz format) and the processed data files (raw counts of mapped sequencing reads) used in this report. All data can be used without restrictions.

## Technical Validation

### Quality of RNA samples

The quality of RNA samples was assessed with an Agilent Bioanalyzer. Most samples showed good RNA Integrity Number (RIN 9.08 ± 1.08, mean ± sd), except for the eight week-2 spleen samples with RIN bellow 5.2. The RNA integrity numbers of all the 320 samples were listed in Online-only Table 2.

### Quality of libraries

**Fig. 2:**
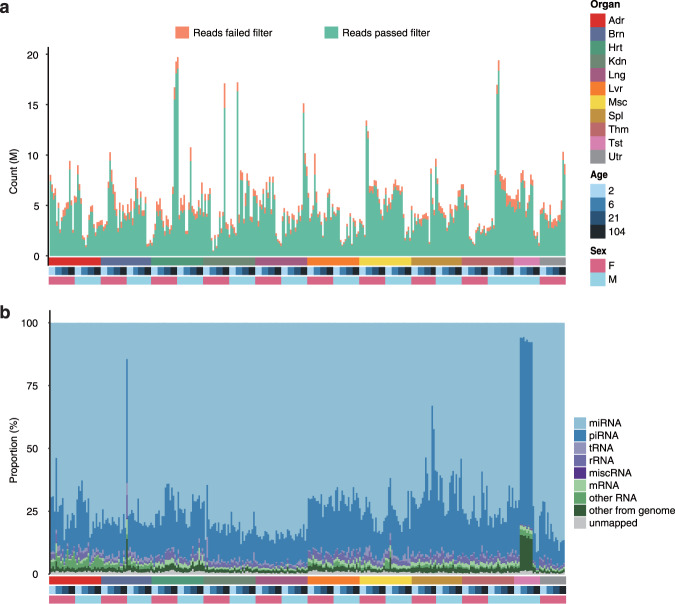
Quality of sequencing reads. (**a**) The overall height of each bar represents the number of total reads sequenced from each RNA sample, and the green part and orange part correspond to the reads which either passed or failed the quality filter, respectively. High-quality reads entered the following mapping workflow. (**b**) Percentages of the reads that mapped to miRNA, piRNA, tRNA, rRNA, miscRNA, mRNA, other RNA, as well as the reads that mapped to none of the aforementioned transcript types but mapped to the rat genome, are represented by different colours. Abbreviation for organs, Adr, adrenal; Brn, brain; Hrt, heart; Kdn, kidney; Lng, lung; Lvr, liver; Msc, skeletal muscle; Spl, spleen; Thm, thymus; Tst, testis; and Utr, uterus.

The quality of miRNA sequencing data for each sample was checked (Fig. 2). Briefly, a total of 1.6 billion raw reads was obtained, with 5.1 million reads per sample on average, which is sufficient for miRNA quantification. After quality filtering and length filtering, 4.5 ± 2.8 million reads of high quality were retained per sample on average (Fig. 2a). The proportions of various transcript types of these reads were demonstrated in Fig. 2b. The majority of libraries were contributed by miRNA and piRNA sequences, followed by tRNA, rRNA, mRNA and other sequences from the rat transcriptome and genome. Most libraries were dominated by miRNA reads (75.6 ± 8.5%), except for the testis samples from the adolescent and adult rats, which were dominated by piRNA (73.8 ± 0.8%). However, the proportion of miRNA for the Brn_F_104_4 sample was 14.4%, much lower than the average proportion of brain samples.

### Reproducibility of biological replicates

**Fig. 3:**
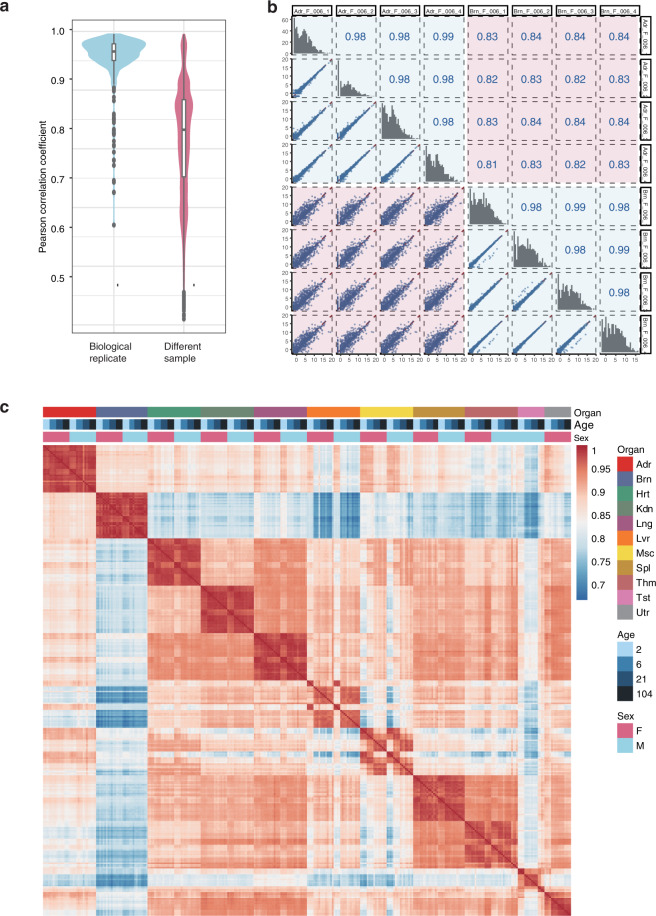
Reproducibility of biological replicates. (**a**) Distributions of the pairwise Pearson correlation coefficients between the biological replicates (navy blue) and between samples from different groups (light blue). (**b**) Correlations between four biological replicates from each of two particular groups (adrenal gland and brain samples of female rats at week 6) were demonstrated as an example. (**c**) Pairwise Pearson correlation coefficients between miRNA expressions of the 318 samples. Abbreviation for organs, Adr, adrenal; Brn, brain; Hrt, heart; Kdn, kidney; Lng, lung; Lvr, liver; Msc, skeletal muscle; Spl, spleen; Thm, thymus; Tst, testis; and Utr, uterus.

**Fig. 4:**
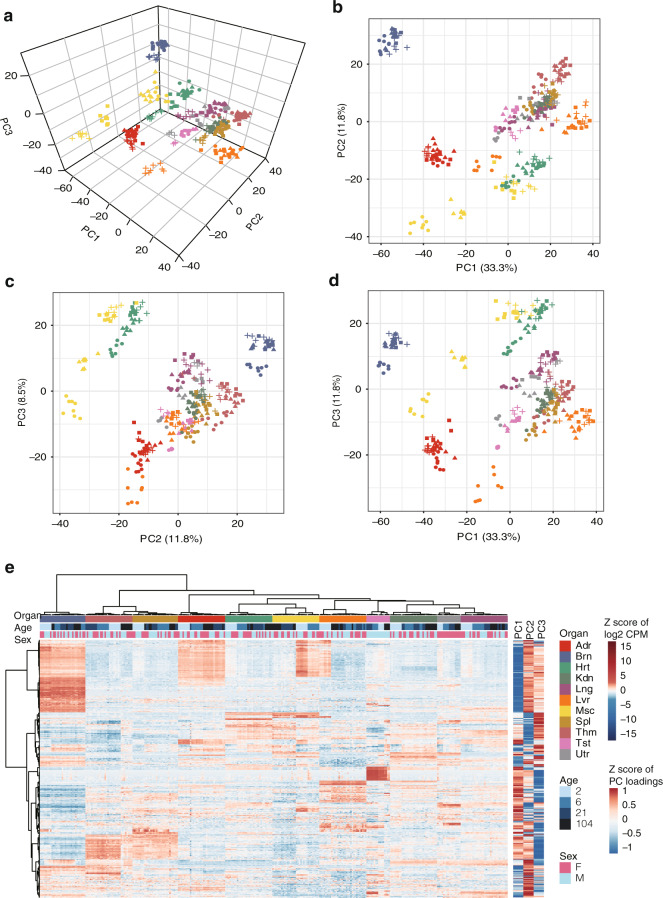
Expression profiles of miRNAs are discriminating among different organs. miRNA expression profiles are depicted by the top three principal components (**a**–**d**). Each point corresponds to one of the 318 samples. Samples from different organs are represented by different colours, and those of different ages are represented by different shapes. (**e**) Hierarchical clustering analysis (HCA) of expression profiles from 318 rat samples with 604 miRNAs. Heatmap (left) for normalized log2 (CPM) values. Right, normalized loadings of each miRNA for the top three PCs. Abbreviation for organs, Adr, adrenal; Brn, brain; Hrt, heart; Kdn, kidney; Lng, lung; Lvr, liver; Msc, skeletal muscle; Spl, spleen; Thm, thymus; Tst, testis; and Utr, uterus.

To assess the reproducibility of biological replicates, we calculated the Pearson’s correlation coefficients between any two of the 320 samples (Fig. 3a–c). The Pearson’s correlation coefficients between miRNA expressions of biological replicates from the same group (0.98 ± 0.01, mean ± sd) were much higher than those from different sample groups (0.8 2 ± 0.09) (Fig. 3a). This result indicated good consistency among biological replicates in which biological variations between animals and technical variations between sample processing were included. Moreover, this conclusion was further confirmed in principal component analysis, from which the majority of biological replicates could be grouped together (Fig. 4a–d).

### Validation of organ specific signature

**Fig. 5:**
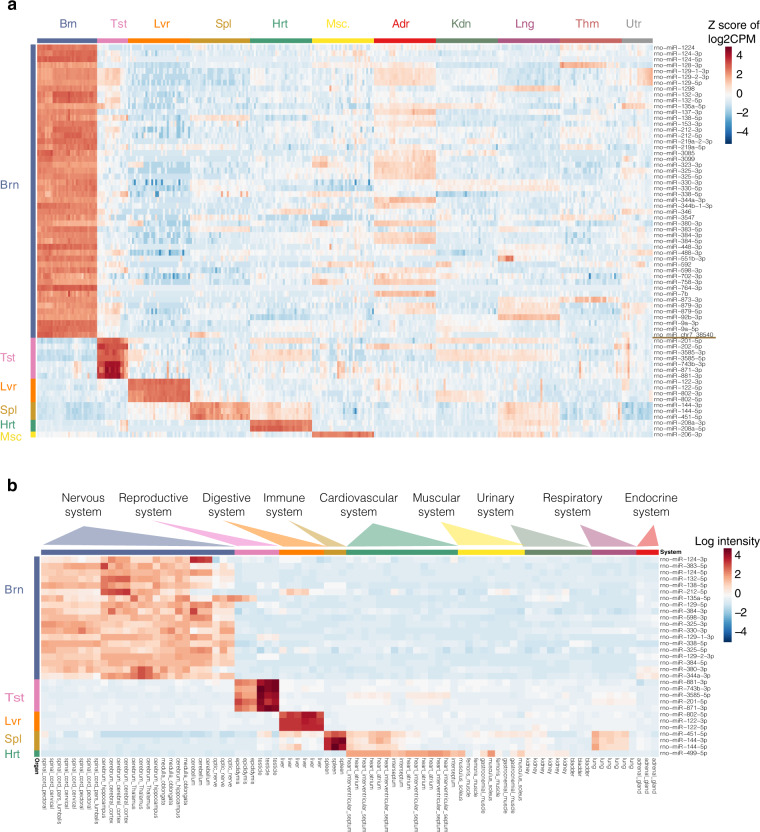
Validation of organ-specific miRNA signatures in a microarray dataset. (**a**) Expression profiles of 67 organ-enriched miRNAs across 318 samples are arranged by organ type. Expression data were Z-score scaled per gene across all 318 samples. Each row represents a miRNA that is enriched in an organ. (**b**) Expression profiles of 31 organ-enriched miRNAs across 23 tissues in the microarray miRNA expression dataset of Minami *et al.*^15^ are arranged by biological system. Each row represents a miRNA that is enriched in an organ. Each column represents a sample profiled in the microarray dataset. Abbreviation for organs, Adr, adrenal; Brn, brain; Hrt, heart; Kdn, kidney; Lng, lung; Lvr, liver; Msc, skeletal muscle; Spl, spleen; Thm, thymus; Tst, testis; and Utr, uterus.

To obtain an overview of miRNA expression profiles, we performed principal component analysis and hierarchical clustering (Fig. 4a–e). The miRNA profiles were discriminating among different organs (Fig. 4e). To identify the miRNAs that contribute the most to organ-specific expression, we set out to identify ‘organ-enriched’ miRNAs. A miRNA is considered ‘organ-enriched’ if it is over-expressed by at least four folds in one organ over any other organs and across all four developmental stages, a quite stringent criterion. Consequently, 67 organ-enriched miRNAs were identified, including a novel miRNA candidate. The z-scaled expression levels of these miRNAs were shown in Fig. 5a.

To independently validate the organ-enriched miRNAs discovered in this study, the literature-reported miRNA profiles of 55 different organs and tissues from normal male rats based on Agilent miRNA microarrays were used^15^. Thirty-one (31) organ-enriched miRNAs identified in our study, including brain-enriched, testis-enriched, liver-enriched, and spleen-enriched and heart-enriched miRNAs, were also profiled in the miRNA microarray dataset and displayed a pattern of high-level expression in biological systems with functions similar to those of the organs examined in this study (Fig. 5b). This result confirmed the reliability of the organ specific signature presented in our dataset.

### Reliability of miRNA-mRNA integration

**Fig. 6:**
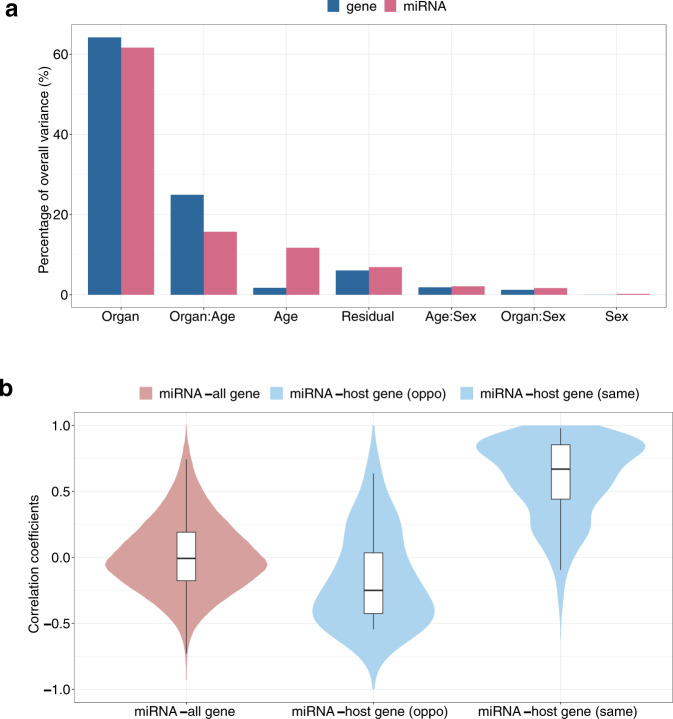
Reliability of miRNA-mRNA integration (**a**) Principal variance component analysis (PVCA) shows the relative contribution of main effects (organ, age, sex, and replicate) and interactions (:) to total model variance in miRNA (blue) and gene (red) expression. Effects in the Y-axis are ordered by proportion of overall variations explained in the miRNA dataset. (**b**) Pairwise correlations between all 10,418,396 miRNA-gene pairs (604 miRNAs versus 17,249 genes), 20 intragenic miRNAs on opposite strand of host genes, 147 intragenic miRNAs on the same strand of host genes.

To examine the reliability of miRNA-mRNA integration using the miRNA dataset presented here and the previously published mRNA dataset, we compared the source of variability in these two datasets. Principal variance component analysis was performed on miRNA and mRNA expression profiles respectively (Fig. 6a). As expected, the ‘organ’ factor accounted for the most total variance in both datasets, with 61.6% for miRNA and 64.2% for mRNA profiles. The variance attributable to each factor was similar in the mRNA and miRNA datasets, except for age. The overall expression difference due to age was significantly higher for miRNA (11.7%) than that for mRNA (1.7%) expression. However, there was a stronger interaction between age and organ type for miRNA (15.7%) compared to that for mRNA (2.5%) expression. It appeared that there is a more dynamic miRNA expression pattern across the four developmental stages independent of organ types, whereas the temporal expression of mRNA appeared to be more organ dependent than that of miRNA expression. These results highlight the concordance of the two datasets.

Meanwhile, the genomic locus of miRNA genes was used to test the reliability of the association between these miRNA and mRNA datasets. We calculated the correlations between expression levels of miRNAs and those of genes (miRNA-gene pairs). The distributions of correlations between several types of miRNA-gene pairs were demonstrated in Fig. 6b. The correlations between all miRNA-gene pairs were weak (0.01 ± 0.28, median ± sd) as expected. Importantly, the co-transcription events of miRNA and host genes were observed in these datasets, as the expressions of intragenic miRNAs were highly positively correlated with those of their host genes on the same strand (0.60 ± 0.29, N = 179). In addition, the slightly negative correlations (−0.17 ± 0.35) between these miRNAs-host genes (opp) pairs confirmed that co-expression events are rare when intragenic miRNAs and the host genes are located on the opposite strands. In summary, the miRNA dataset along with the mRNA datasets provided a reliable and unique resource for integration.

## Data Availability

The code required for reproducing the figures and tables is freely available on GitHub (https://github.com/XintongYao-96/Rat-microRNA-Bodymap).

## Online-only Table

**Online Only Table 1:** Novel miRNA candidates.

miRNA_ID	miRDeep2_score	mature_seq	star_seq	pre_seq	position	piRNA_alert	Organ_enriched	Adr_F_002_1	Adr_F_002_2	Adr_F_002_3	Adr_F_002_4	Adr_F_006_1	Adr_F_006_2	Adr_F_006_3	Adr_F_006_4	Adr_F_021_1	Adr_F_021_2	Adr_F_021_3	Adr_F_021_4	Adr_F_104_1	Adr_F_104_2	Adr_F_104_3	Adr_F_104_4	Adr_M_002_1	Adr_M_002_2	Adr_M_002_3	Adr_M_002_4	Adr_M_006_1	Adr_M_006_2	Adr_M_006_3	Adr_M_006_4	Adr_M_021_1	Adr_M_021_2	Adr_M_021_3	Adr_M_021_4	Adr_M_104_1	Adr_M_104_2	Adr_M_104_3	Adr_M_104_4	Brn_F_002_1	Brn_F_002_2	Brn_F_002_3	Brn_F_002_4	Brn_F_006_1	Brn_F_006_2	Brn_F_006_3	Brn_F_006_4	Brn_F_021_1	Brn_F_021_2	Brn_F_021_3	Brn_F_021_4	Brn_F_104_1	Brn_F_104_2	Brn_F_104_3	Brn_F_104_4	Brn_M_002_1	Brn_M_002_2	Brn_M_002_3	Brn_M_002_4	Brn_M_006_1	Brn_M_006_2	Brn_M_006_3Brn_M_006_4 Brn_M_021_1 Brn_M_021_2 Brn_M_021_3 Brn_M_021_4 Brn_M_104_1 Brn_M_104_2 Brn_M_104_3 Brn_M_104_4 Hrt_F_002_1 Hrt_F_002_2 Hrt_F_002_3 Hrt_F_002_4 Hrt_F_006_1 Hrt_F_006_2 Hrt_F_006_3 Hrt_F_006_4 Hrt_F_021_1 Hrt_F_021_2 Hrt_F_021_3 Hrt_F_021_4 Hrt_F_104_1 Hrt_F_104_2 Hrt_F_104_3 Hrt_F_104_4 Hrt_M_002_1 Hrt_M_002_2 Hrt_M_002_3 Hrt_M_002_4 Hrt_M_006_1 Hrt_M_006_2 Hrt_M_006_3 Hrt_M_006_4 Hrt_M_021_1 Hrt_M_021_2 Hrt_M_021_3 Hrt_M_021_4 Hrt_M_104_1 Hrt_M_104_2 Hrt_M_104_3 Hrt_M_104_4 Kdn_F_002_1 Kdn_F_002_2 Kdn_F_002_3 Kdn_F_002_4 Kdn_F_006_1 Kdn_F_006_2 Kdn_F_006_3 Kdn_F_006_4 Kdn_F_021_1 Kdn_F_021_2 Kdn_F_021_3 Kdn_F_021_4 Kdn_F_104_1 Kdn_F_104_2 Kdn_F_104_3 Kdn_F_104_4 Kdn_M_002_1 Kdn_M_002_2 Kdn_M_002_3 Kdn_M_002_4 Kdn_M_006_1 Kdn_M_006_2 Kdn_M_006_3 Kdn_M_006_4 Kdn_M_021_1 Kdn_M_021_2 Kdn_M_021_3 Kdn_M_021_4 Kdn_M_104_1 Kdn_M_104_2 Kdn_M_104_3 Kdn_M_104_4 Lng_F_002_1 Lng_F_002_2 Lng_F_002_3 Lng_F_002_4 Lng_F_006_1 Lng_F_006_2 Lng_F_006_3 Lng_F_006_4 Lng_F_021_1 Lng_F_021_2 Lng_F_021_3 Lng_F_021_4 Lng_F_104_1 Lng_F_104_2 Lng_F_104_3 Lng_F_104_4 Lng_M_002_1 Lng_M_002_2 Lng_M_002_3 Lng_M_002_4 Lng_M_006_1 Lng_M_006_2 Lng_M_006_3 Lng_M_006_4 Lng_M_021_1 Lng_M_021_2 Lng_M_021_3 Lng_M_021_4 Lng_M_104_1 Lng_M_104_2 Lng_M_104_3 Lng_M_104_4 Lvr_F_002_1 Lvr_F_002_2 Lvr_F_002_3 Lvr_F_002_4 Lvr_F_006_1 Lvr_F_006_2 Lvr_F_006_3 Lvr_F_006_4 Lvr_F_021_1 Lvr_F_021_2 Lvr_F_021_3 Lvr_F_021_4 Lvr_F_104_1 Lvr_F_104_2 Lvr_F_104_3 Lvr_F_104_4 Lvr_M_002_1 Lvr_M_002_2 Lvr_M_002_3 Lvr_M_002_4 Lvr_M_006_1 Lvr_M_006_2 Lvr_M_006_3 Lvr_M_006_4 Lvr_M_021_1 Lvr_M_021_2 Lvr_M_021_3 Lvr_M_021_4 Lvr_M_104_1 Lvr_M_104_2 Lvr_M_104_3 Lvr_M_104_4 Msc_F_002_1 Msc_F_002_2 Msc_F_002_3 Msc_F_002_4 Msc_F_006_1 Msc_F_006_2 Msc_F_006_3 Msc_F_006_4 Msc_F_021_1 Msc_F_021_2 Msc_F_021_3 Msc_F_021_4 Msc_F_104_1 Msc_F_104_2 Msc_F_104_3 Msc_F_104_4 Msc_M_002_1 Msc_M_002_2 Msc_M_002_3 Msc_M_002_4 Msc_M_006_1 Msc_M_006_2 Msc_M_006_3 Msc_M_006_4 Msc_M_021_1 Msc_M_021_2 Msc_M_021_3 Msc_M_021_4 Msc_M_104_1 Msc_M_104_2 Msc_M_104_3 Msc_M_104_4 Spl_F_002_1 Spl_F_002_2 Spl_F_002_3 Spl_F_002_4 Spl_F_006_1 Spl_F_006_2 Spl_F_006_3 Spl_F_006_4 Spl_F_021_1 Spl_F_021_2 Spl_F_021_3 Spl_F_021_4 Spl_F_104_1 Spl_F_104_2 Spl_F_104_3 Spl_F_104_4 Spl_M_002_1 Spl_M_002_2 Spl_M_002_3 Spl_M_002_4 Spl_M_006_1 Spl_M_006_2 Spl_M_006_3 Spl_M_006_4 Spl_M_021_1 Spl_M_021_2 Spl_M_021_3 Spl_M_021_4 Spl_M_104_1 Spl_M_104_2 Spl_M_104_3 Spl_M_104_4 Thm_F_002_1 Thm_F_002_2 Thm_F_002_3 Thm_F_002_4 Thm_F_006_1 Thm_F_006_2 Thm_F_006_3 Thm_F_006_4 Thm_F_021_1 Thm_F_021_2 Thm_F_021_3 Thm_F_021_4 Thm_F_104_1 Thm_F_104_2 Thm_F_104_3 Thm_F_104_4 Thm_M_002_1 Thm_M_002_2 Thm_M_002_3 Thm_M_002_4 Thm_M_006_1 Thm_M_006_2 Thm_M_006_3 Thm_M_006_4 Thm_M_021_1 Thm_M_021_2 Thm_M_021_3 Thm_M_021_4 Thm_M_104_1 Thm_M_104_2 Thm_M_104_3 Thm_M_104_4 Tst_M_002_1 Tst_M_002_2 Tst_M_002_3 Tst_M_002_4 Tst_M_006_1 Tst_M_006_2 Tst_M_006_3 Tst_M_006_4 Tst_M_021_1 Tst_M_021_2 Tst_M_021_3 Tst_M_021_4 Tst_M_104_1 Tst_M_104_2 Tst_M_104_3 Tst_M_104_4 Utr_F_002_1 Utr_F_002_2 Utr_F_002_3 Utr_F_002_4 Utr_F_006_1 Utr_F_006_2 Utr_F_006_3 Utr_F_006_4 Utr_F_021_1 Utr_F_021_2 Utr_F_021_3 Utr_F_021_4 Utr_F_104_1 Utr_F_104_2 Utr_F_104_3 Utr_F_104_4
rno_miR_chr10_8434	210	UCAUGUGGAAGCCUUGCUAAGC	GAGGCAAGGCUGCCGCAUGGCUA	GAGGCAAGGCUGCCGCAUGGCUAUGAAGCGGAAGGUCAUGUGGAAGCCUUGCUAAGC	CHR10:92664597..92664654:-	NA	Msc	0	0	0	0	0	0	0	0	0	0	0	0	0	0	0	0	0	0	0	0	0	0	0	0	0	0	0	0	0	0	0	0	0	0	0	0	0	0	0	0	0	0	0	0	0	0	0	1	0	0	0	0	0	0	40 0 0 0 0 0 0 0 0 0 0 0 0 0 0 0 0 0 0 0 0 0 0 0 0 0 0 0 0 0 0 0 0 0 0 0 0 1 0 0 0 0 0 0 0 1 0 0 0 0 0 0 0 0 0 0 0 0 0 0 0 0 0 0 0 0 0 0 0 0 0 0 0 3 0 0 1 0 0 0 0 0 2 0 0 0 0 0 0 0 0 0 0 0 1 0 0 0 0 1 0 0 0 0 0 0 0 0 0 0 0 0 0 0 0 0 0 0 0 0 0 0 0 0 0 0 0 0 0 0 0 0 1 0 0 0 0 11 9 2 10 11 5 17 6 7 7 14 24 21 11 37 17 17 6 8 2 8 7 11 8 19 7 5 22 28 12 9 18 0 0 0 0 0 0 0 0 0 0 0 0 0 0 0 3 0 0 0 0 0 0 0 0 0 0 0 0 0 0 0 0 0 0 0 0 0 0 0 0 0 0 0 0 0 0 0 0 1 0 0 0 0 0 0 0 0 0 0 0 0 0 0 0 0 0 0 0 0 0 0 0 0 0 0 0 0 0 0 0 1 0 0 0 3 0 0 0 0 1 0 0 0 0 0 0
rno_miR_chr12_11512	600	UGGGCACUUAUUUCCUGACAGA	AGUCAGGACAUUGUGACCAGGA	AGUCAGGACAUUGUGACCAGGAAGUACUUUGCAUGGUUCUGGGCACUUAUUUCCUGACAGA	CHR12:38528049..38528110:-	NA	Hrt	2	3	2	0	3	0	0	1	0	2	2	1	2	1	0	0	2	1	0	2	1	1	1	0	1	1	0	0	1	2	0	1	1	5	3	0	5	4	0	5	9	2	4	12	2	4	7	2	1	1	0	1	4	5	92 1 5 1 7 9 3 5 0 14 9 7 14 6 12 18 11 23 19 26 16 20 28 50 21 5 8 13 14 5 29 20 15 23 20 31 12 13 31 11 32 1 2 3 4 6 3 4 7 3 4 3 4 1 7 1 1 3 1 2 5 6 3 4 4 3 3 0 4 1 3 5 3 1 2 1 0 1 0 0 1 5 3 1 2 0 4 1 2 0 0 0 3 1 1 2 0 2 1 0 2 3 3 4 2 0 0 0 0 0 0 0 0 0 0 0 1 0 0 0 0 0 0 0 0 0 0 0 0 0 0 0 0 0 0 0 0 2 0 0 0 0 0 0 0 0 0 0 0 0 0 1 0 0 0 0 0 0 0 0 0 0 1 0 0 0 0 0 0 0 1 1 0 1 1 1 0 0 1 1 2 1 0 0 8 0 0 1 0 3 1 1 1 0 0 2 1 1 0 1 0 1 2 0 1 1 0 3 2 0 0 0 2 0 1 1 0 1 0 0 0 1 1 2 0 2 1 1 0 1 0 0 2 0 0 3 2 0 0 0 2 0 0 0 2 0 1 0 0 0 1 0 0 0 0 0 0 0 0 1 0 1 1 0 0
rno_miR_chr19_20934	310	UCUCUCUUCCUCCUCACUGUGGA	CACGGAUAGGAGGAAGUGAGCU	CACGGAUAGGAGGAAGUGAGCUGAGUACCAUGUUCAUCUCUCUCUUCCUCCUCACUGUGGA	CHR19:53760883..53760944:-	piR-rno-676178	Brn	0	0	0	0	0	0	0	0	0	1	0	0	0	0	1	0	0	0	0	0	2	0	0	1	0	0	0	0	0	0	0	0	1	7	9	7	4	9	4	9	3	17	1	8	0	3	7	3	5	6	6	6	8	12	61 9 5 4 8 2 3 6 9 0 0 0 0 0 0 0 1 0 0 0 0 0 0 0 0 0 0 0 0 0 0 0 0 1 0 1 0 0 0 0 0 0 0 0 1 0 0 0 1 1 0 0 0 1 0 0 1 1 1 0 2 0 1 0 2 0 1 0 1 0 0 0 1 4 0 2 1 2 1 5 0 0 0 2 1 1 1 1 1 2 2 0 1 5 3 4 0 1 2 3 0 1 0 3 1 0 0 0 0 0 0 0 0 0 0 0 0 1 0 0 0 0 0 0 0 0 0 0 0 0 0 0 0 0 0 0 0 0 0 0 0 0 0 0 0 0 0 0 0 0 0 0 0 0 0 0 0 0 0 0 0 0 0 0 0 0 1 1 0 0 0 1 0 0 0 0 0 0 0 0 0 0 0 1 5 0 0 0 0 0 0 0 0 0 0 0 0 2 1 0 1 0 0 0 1 0 0 0 1 0 0 1 1 0 1 0 1 0 0 1 1 0 1 0 0 0 0 0 2 0 0 1 0 2 1 4 1 1 1 1 1 0 3 0 0 0 0 0 0 0 1 1 1 0 1 0 2 0 2 2 1 1 0 0 0
rno_miR_chr1_3858	940	CCUCCCUGUCUCCUCCCUGCAG	CUCAGGGAGGGGACACGGAGUGC	CUCAGGGAGGGGACACGGAGUGCAGGCACUCACCACUGGCCCUCCCUGUCUCCUCCCUGCAG	CHR1:84368875..84368937:-	piR-rno-2470659	Msc	0	0	0	0	0	0	0	0	0	0	0	0	0	0	0	0	0	0	0	2	0	0	0	0	0	1	0	0	0	0	0	0	0	1	0	0	6	3	0	1	0	0	0	1	0	0	1	0	0	0	0	1	0	0	50 0 1 0 1 2 0 1 0 0 0 0 0 0 0 0 1 0 0 0 0 0 0 0 0 0 0 0 0 0 0 0 0 0 0 0 0 1 0 1 0 0 0 0 0 0 1 2 1 0 0 0 1 0 0 0 0 0 1 0 0 1 0 1 0 0 0 1 0 0 1 0 1 1 0 1 3 0 0 1 3 0 2 1 0 0 0 0 0 0 0 0 0 1 0 0 0 0 1 1 0 2 0 0 0 0 0 0 0 0 0 0 0 0 0 0 0 0 0 0 0 0 0 0 0 0 0 0 0 0 1 1 0 0 1 0 0 11 19 7 28 11 26 14 10 20 18 11 19 31 25 45 35 19 13 16 6 18 13 23 10 26 20 6 34 42 23 28 43 0 1 0 0 0 0 0 0 1 0 0 0 0 0 0 11 0 0 2 0 0 2 0 0 0 0 0 0 0 0 2 0 0 0 1 0 0 1 1 0 0 0 1 0 0 0 0 0 0 0 0 1 0 1 0 0 0 0 0 0 0 1 0 1 0 0 0 1 0 0 0 0 0 0 0 0 0 0 0 0 0 0 0 0 0 0 0 0 0 0 0 0 0 0 0 0
rno_miR_chr20_23611	2400	UGUGCCCUGUUCCCCCUCCCUGC	UGGGGGGUGGGAGGGGCAUCAGC	UGGGGGGUGGGAGGGGCAUCAGCUCCUAUUCACCGGUGCUGUGCCCUGUUCCCCCUCCCUGC	CHR20:4028583..4028645:+	NA	Adr	80	110	78	91	106	74	151	227	124	209	110	168	174	118	80	123	137	105	71	121	244	96	141	163	127	109	44	145	116	168	215	73	0	1	0	1	1	0	0	0	0	0	0	1	0	0	1	3	0	1	0	0	0	0	220 1 0 0 1 1 0 1 0 0 0 0 1 0 0 0 1 0 0 0 0 0 0 0 0 0 0 0 0 2 0 0 0 0 0 0 0 0 0 0 0 0 0 0 0 0 0 0 0 0 1 0 0 0 0 0 0 0 0 0 0 0 0 0 0 0 0 0 0 0 0 0 0 0 0 0 1 0 0 1 1 0 0 0 0 0 1 1 1 0 0 0 0 0 0 0 0 1 0 0 0 0 0 0 0 0 0 0 0 0 0 0 3 0 0 0 1 0 0 0 0 0 0 0 0 0 0 0 0 0 0 0 0 0 1 0 0 0 0 0 0 0 0 0 0 0 0 0 0 0 0 1 0 0 0 0 1 2 2 1 0 0 0 0 0 0 0 0 0 0 0 0 0 0 1 0 0 2 0 0 0 0 0 0 18 0 1 0 0 0 0 0 0 0 1 0 0 0 0 0 0 0 0 0 0 0 0 0 1 0 0 1 0 0 1 0 0 1 0 0 0 1 0 0 0 0 0 0 1 0 0 0 0 1 0 1 1 0 0 0 0 0 0 1 0 0 0 0 0 0 0 0 0 0 0 0 1 1 0 0 0 1 0 0 0
rno_miR_chr3_28394	310	AUAUAGAGGAUAAUAUAAAUGU	AUUUAUAUUAUCCUCUAUAUGU	AUUUAUAUUAUCCUCUAUAUGUAUAUAUAUGUAUAAUAUAGAGGAUAAUAUAAAUGU	CHR3:36690295..36690352:-	NA	Msc	0	0	0	0	0	0	0	0	0	0	0	1	0	0	0	0	0	0	0	0	0	0	0	0	0	0	0	0	0	0	0	0	0	0	0	0	0	0	0	0	0	0	0	0	0	0	0	0	0	0	0	0	0	0	00 0 0 0 0 0 0 1 0 0 1 0 0 0 0 1 0 1 0 1 0 0 0 1 1 0 0 0 0 0 0 0 0 0 1 2 0 0 2 0 1 0 0 0 0 0 0 0 0 0 0 0 0 0 0 0 0 0 0 0 0 0 0 0 0 0 0 0 0 0 0 1 0 2 0 0 1 7 1 2 0 3 1 2 0 0 2 0 0 0 0 0 2 1 2 1 0 1 1 5 4 2 2 5 1 0 0 0 0 0 0 0 0 0 0 0 0 0 0 0 0 0 0 0 0 0 0 2 0 0 0 0 0 0 0 0 0 8 3 1 10 13 11 9 11 19 9 7 14 23 10 39 23 6 5 8 4 18 14 21 13 25 5 3 28 27 13 36 22 0 0 0 0 0 0 0 0 0 0 0 0 0 0 0 6 0 0 0 0 0 0 0 0 0 0 0 0 0 0 0 0 0 0 0 0 0 0 0 1 0 0 0 0 0 0 0 0 1 0 0 0 0 0 0 0 0 0 1 0 0 1 0 0 1 0 0 0 0 0 0 0 0 0 0 0 0 0 0 0 0 0 0 0 0 0 0 0 0 0 1 0 0 0 0 0
rno_miR_chr5_34126	1800	CACAGCGGAGCUGGGCACUGGCGU	CCACCCCUGGCUCUGCUGGAU	CACAGCGGAGCUGGGCACUGGCGUCACGCAGUCCUUGUUGCCACCCCUGGCUCUGCUGGAU	CHR5:149874651..149874712:+	NA	Brn	1	2	1	2	1	1	3	0	1	1	1	2	2	1	3	0	2	1	4	1	1	0	0	1	0	4	1	0	0	3	0	0	82	128	92	129	132	112	86	132	87	106	66	97	37	82	93	14	84	125	132	85	74	92	8891 77 89 72 105 123 94 73 81 2 1 0 0 0 0 1 1 0 2 0 0 4 1 4 5 1 1 1 2 0 3 1 0 1 0 2 2 0 2 4 0 1 5 4 3 2 1 3 6 2 1 2 0 4 1 2 2 3 1 5 3 2 2 0 3 1 2 1 2 1 1 1 4 1 1 0 0 0 0 0 1 1 0 0 1 2 1 0 0 0 2 1 2 0 2 0 0 1 2 0 0 0 0 1 0 0 1 0 0 0 0 0 0 0 0 0 0 0 0 0 0 0 0 0 0 0 0 0 0 0 0 0 0 0 0 0 0 0 0 0 0 0 1 0 0 1 1 0 1 0 0 0 1 0 0 0 2 1 0 0 0 0 1 0 1 0 0 0 0 0 1 3 0 1 1 1 0 1 0 0 1 0 0 0 20 0 1 0 2 0 1 0 0 1 0 1 1 1 2 0 0 0 0 0 1 0 0 0 0 0 1 1 2 0 0 1 0 0 0 1 0 0 2 0 0 1 1 0 1 0 0 0 1 1 0 3 2 0 0 0 1 0 0 0 0 1 1 2 0 3 7 0 3 3 0 1 0 6 1 2 3 2 0 4 0
rno_miR_chr7_38540	8000	UUUCCUCUCUGCCCCAUAGGGUG	CCUCUAGGGAAGAGAAGGUUGG	UUUCCUCUCUGCCCCAUAGGGUGUAGCUCUAACUACCCUCUAGGGAAGAGAAGGUUGG	CHR7:36786031..36786089:+	NA	Brn	1	2	0	2	4	1	3	3	1	2	0	1	0	0	0	1	2	2	2	1	0	0	1	0	0	0	0	0	0	1	0	0	516	785	774	720	484	499	327	660	357	418	224	561	134	223	394	77	619	1033	536	545	493	579	391262 324 388 232 372 440 333 365 216 0 2 0 0 2 0 5 0 1 0 1 0 0 1 0 1 2 1 2 0 0 1 1 0 0 0 1 0 0 0 0 0 1 1 2 4 2 0 0 1 0 0 0 0 0 0 0 0 8 10 7 3 1 0 0 0 0 0 0 0 0 0 0 0 3 4 1 2 1 0 0 1 1 0 0 0 0 1 2 1 3 1 0 4 0 2 0 0 0 0 2 0 1 0 0 0 0 0 0 0 0 0 0 0 0 0 0 0 0 0 0 1 0 0 0 0 0 0 0 0 0 0 0 1 0 0 0 0 7 0 1 3 1 0 1 0 1 0 1 0 0 1 0 0 5 3 2 4 0 0 1 0 0 1 1 1 0 0 0 1 31 11 22 26 9 4 1 4 1 1 0 1 0 0 0 116 25 35 34 13 6 6 8 3 0 0 1 1 0 1 0 0 1 1 1 0 0 0 0 0 0 0 0 0 0 0 0 2 1 0 3 3 0 0 0 0 1 0 1 0 0 0 0 0 8 5 7 6 1 1 0 1 0 2 1 0 0 0 0 1 10 10 6 5 3 2 0 1 1 1 1 2 0 0 1 0
rno_miR_chr7_40035	1300	ACUCUAGCUGCCAAAGGCGCU	CGCUUUGCUCAGCCAGUGUAGA	ACUCUAGCUGCCAAAGGCGCUUCUCCUUCUGAACAGAGCGCUUUGCUCAGCCAGUGUAGA	CHR7:26676114..26676174:-	piR-rno-2374001	Brn	1	2	1	2	1	1	0	2	1	1	3	2	0	0	1	1	0	0	0	1	1	0	1	1	1	1	0	0	1	1	0	0	126	144	131	123	67	62	49	83	74	110	51	78	26	43	58	14	161	162	88	95	78	110	8036 40 53 58 46 51 47 51 25 0 0 0 0 0 0 0 0 0 0 0 0 0 0 0 0 0 0 0 0 0 1 0 0 0 0 0 0 0 0 0 0 0 1 4 1 0 0 0 0 0 0 0 0 0 0 0 0 5 4 3 2 0 0 2 0 1 1 0 0 0 2 0 0 3 0 0 0 0 0 0 0 0 0 0 0 0 0 0 0 2 0 1 2 0 0 0 0 0 0 0 0 0 1 0 0 0 0 0 1 0 0 0 0 0 0 0 0 0 0 0 0 0 0 0 0 0 0 0 0 0 0 0 0 0 0 0 0 0 1 0 0 0 0 0 0 0 0 0 0 0 0 0 0 0 1 1 0 0 1 0 0 0 0 0 0 0 0 0 0 0 0 0 0 1 0 0 1 0 0 0 0 0 0 0 16 1 0 1 0 0 0 0 0 0 0 0 0 1 0 0 0 1 0 1 0 0 0 0 0 0 0 0 0 0 0 0 0 0 0 0 0 0 0 0 0 0 0 0 1 0 1 0 1 15 2 25 9 1 0 0 1 0 1 0 0 1 0 0 0 5 14 1 5 2 0 1 2 3 4 1 3 0 1 1 0
rno_miR_chrX_48159	1100	CAAAUCUUAUUUGAGCACCUGU	AGGUCCUCAGUAAGUAUUUGUU	AGGUCCUCAGUAAGUAUUUGUUGAAAAAAUAAAUGAACCAACAAAUCUUAUUUGAGCACCUGU	CHRX:110687149..110687212:+	NA	Brn	0	0	0	0	0	0	0	0	0	0	0	0	0	0	0	0	0	0	0	0	0	0	0	0	0	0	0	0	0	0	1	0	35	61	25	46	77	54	28	93	43	53	29	59	18	15	39	13	36	33	20	28	54	46	5131 31 65 43 54 49 42 48 32 0 0 0 0 0 0 1 0 0 0 0 0 0 1 0 0 0 0 0 0 0 0 0 0 0 0 0 0 0 0 0 1 0 0 0 0 0 0 0 0 0 0 0 0 0 0 0 0 0 0 0 0 0 0 0 0 0 0 0 0 0 0 0 0 1 0 1 2 3 0 1 1 1 0 1 1 2 1 0 0 2 0 0 1 4 1 2 0 0 1 1 1 1 0 0 0 0 0 0 0 1 0 0 0 0 0 0 0 0 0 0 0 0 0 0 0 0 0 0 0 0 0 0 0 0 0 0 0 0 0 0 0 0 2 0 0 0 0 0 0 0 0 0 0 0 0 0 0 0 0 0 0 0 0 0 0 0 0 1 0 0 0 0 0 0 0 0 0 0 0 0 0 0 0 0 5 0 0 0 0 0 0 0 0 0 0 0 0 0 0 0 0 0 0 1 0 0 0 1 0 0 1 0 0 0 1 0 0 0 0 1 0 0 1 0 0 0 0 0 0 0 0 0 1 0 0 0 0 0 0 0 1 0 0 0 0 1 0 0 0 0 0 0 0 0 1 0 0 0 0 0 0 0 0 0 0
rno_miR_chrX_48468	3900	UGACUUUCCAGAGAACCCUUGA	AAGGGUUCUCUGGAAAGUCGGU	AAGGGUUCUCUGGAAAGUCGGUUGUGUUGGAUGGUGUUUUGCUGACUUUCCAGAGAACCCUUGA	CHRX:35090149..35090213:-	NA	Thm	14	7	2	12	6	3	6	7	5	3	4	8	9	10	5	3	8	6	7	11	0	7	5	3	5	5	2	4	6	5	7	2	6	6	3	3	6	1	3	6	13	6	5	10	2	3	8	4	4	8	7	9	1	1	469 4 9 5 7 13 6 5 9 7 6 3 2 2 3 7 2 8 4 3 5 4 4 18 9 4 10 7 9 5 4 5 3 5 3 8 3 3 1 7 3 8 8 11 13 3 5 3 8 3 6 2 2 1 5 15 10 8 7 9 9 6 5 3 1 3 1 5 7 2 18 7 12 23 16 6 9 19 10 9 19 25 4 0 11 0 25 5 8 9 25 7 16 27 16 14 2 18 9 10 6 23 24 20 20 2 2 0 1 2 0 0 0 0 0 1 2 0 0 0 2 0 0 2 1 0 2 1 1 0 0 0 0 0 3 1 3 6 5 0 4 5 0 0 0 0 1 0 1 0 2 2 0 4 5 5 4 3 0 3 1 0 0 1 0 0 2 3 0 32 31 56 28 43 33 42 69 27 66 32 26 25 29 39 132 18 13 35 8 49 61 22 27 59 88 61 79 65 79 33 27 122 140 117 106 151 64 115 302 122 194 74 115 27 144 54 151 51 160 60 49 105 126 129 61 56 190 36 142 16 73 27 67 2 1 2 7 2 9 1 1 5 5 8 2 0 2 2 2 9 14 6 10 9 13 9 6 23 25 12 9 15 8 7 6
rno_miR_chrX_48878	5500	GUUGGCACUUUCUUGAGUGAGG	CACUCCAAAAGUGCCAAUCAG	CACUCCAAAAGUGCCAAUCAGGAUAACUAAAAUAUGGUUGGCACUUUCUUGAGUGAGG	CHRX:146675517..146675575:-	piR-rno-317792	Tst	2	1	0	4	0	1	1	0	3	1	0	0	1	0	0	0	0	0	0	1	0	1	1	3	1	0	1	3	0	0	0	0	1	0	0	2	0	1	0	0	1	2	0	1	0	1	0	0	0	2	0	0	0	1	1710 0 12 3 4 0 1 1 0 15 21 4 9 3 15 15 6 20 2 11 9 9 7 33 7 10 3 5 17 13 18 11 10 11 13 17 5 8 5 30 11 8 10 18 13 21 24 12 12 2 11 4 7 5 6 11 9 13 9 7 11 15 3 4 12 4 1 3 24 3 10 7 4 20 7 5 10 17 2 9 16 20 10 6 24 9 13 4 5 8 11 2 11 20 5 16 5 13 10 6 9 1 7 7 2 1 1 5 4 4 1 0 2 0 0 2 0 0 0 0 1 5 8 7 2 2 0 0 1 0 0 0 0 1 1 0 0 0 1 0 2 1 0 1 0 0 0 0 0 0 0 0 0 2 2 2 0 0 2 1 1 0 1 0 1 0 1 1 0 2 0 1 0 1 0 0 0 1 0 1 1 1 0 5 324 0 0 1 0 0 1 0 0 0 1 0 3 1 1 0 0 1 1 0 0 0 0 3 0 0 0 0 0 1 0 1 1 0 0 0 0 0 1 1 0 0 0 0 0 0 1 0 1 739 320 930 1329 603 701 136 249 2222 1352 391 363 177 245 39 56 4 5 6 10 3 0 0 1 2 2 4 3 0 0 3 0

**Online Only Table 2:** Metadata.

Sample_ID	Colnames.miRNA	RNA_Sample_ID	RNA_A260.A280_Ratio	RNA_RIN	Organ	Organ_Abbr_2chars	Organ_Abbr_3chars	Sex	Age	BioRep	Group	Total_forAlign	Total_sequence	mRNA	miRNA	miscRNA	other_RNA	other_from_Genome	piRNA	rRNA	tRNA	total_genome	unmapped	GEO_Accession	SRA_ID
Adr_F_002_1	lane3_Adr_F_2_1_1	130	1.97	9.3	Adrenal	Ad	Adr	F	2	1	Adr-F-2	4500386	5756631	41234	3116039	1806	115281	65806	982384	97782	35361	4400075	44693	GSM5251160	SRR14265264
Adr_F_002_2	lane4_Adr_F_2_2_1	138	1.99	9.1	Adrenal	Ad	Adr	F	2	2	Adr-F-2	4940517	6246771	71007	3401988	2612	85221	103312	1078266	82158	55825	4816253	60128	GSM5251161	SRR14265265
Adr_F_002_3	lane5_Adr_F_2_3_1	146	2	9.5	Adrenal	Ad	Adr	F	2	3	Adr-F-2	3426021	3758223	19631	2567606	1013	111177	43606	558636	77807	15298	3364020	31247	GSM5251162	SRR14265266
Adr_F_002_4	lane2_Adr_F_2_4_1	154	1.99	8.5	Adrenal	Ad	Adr	F	2	4	Adr-F-2	4562277	6380690	176766	2445924	6058	154689	171538	1301680	165069	71648	4434302	68905	GSM5251163	SRR14265267
Adr_F_006_1	lane8_Adr_F_6_1_1	132	1.99	9.4	Adrenal	Ad	Adr	F	6	1	Adr-F-6	6623262	7932484	45498	4970220	2170	241994	112203	901747	115692	137584	6456917	96154	GSM5251164	SRR14265268
Adr_F_006_2	lane2_Adr_F_6_2_1	140	2.02	9.3	Adrenal	Ad	Adr	F	6	2	Adr-F-6	2354139	2778635	17298	1710772	742	102783	35393	368134	45951	50830	2309903	22236	GSM5251165	SRR14265269
Adr_F_006_3	lane3_Adr_F_6_3_1	148	2.02	9.5	Adrenal	Ad	Adr	F	6	3	Adr-F-6	4289563	5074118	41225	3100241	1846	89294	64020	829258	77863	44780	4208613	41036	GSM5251166	SRR14265270
Adr_F_006_4	lane8_Adr_F_6_4_1	156	2.02	8.9	Adrenal	Ad	Adr	F	6	4	Adr-F-6	6544716	7583121	69965	4554036	3011	171235	114796	1320159	161488	63175	6384305	86851	GSM5251167	SRR14265271
Adr_F_021_1	lane7_Adr_F_21_1_1	134	1.89	9.5	Adrenal	Ad	Adr	F	21	1	Adr-F-21	7126641	7712135	24874	6150983	1158	109445	80101	605226	46579	48064	7015703	60211	GSM5251168	SRR14265272
Adr_F_021_2	lane6_Adr_F_21_2_1	142	1.93	9.5	Adrenal	Ad	Adr	F	21	2	Adr-F-21	7021446	7540256	28272	5748342	1275	244438	99768	711267	62356	45520	6880639	80208	GSM5251169	SRR14265273
Adr_F_021_3	lane2_Adr_F_21_3_1	150	1.89	9.5	Adrenal	Ad	Adr	F	21	3	Adr-F-21	3964350	4668361	18109	3018220	920	255300	73714	453354	44636	44857	3876221	55240	GSM5251170	SRR14265274
Adr_F_021_4	lane6_Adr_F_21_4_1	158	1.89	9	Adrenal	Ad	Adr	F	21	4	Adr-F-21	6886896	7470341	39012	5625549	1655	207404	105741	724924	52680	46547	6737308	83384	GSM5251171	SRR14265275
Adr_F_104_1	lane6_Adr_F_100_1_1	136	2.01	9.5	Adrenal	Ad	Adr	F	104	1	Adr-F-104	6207777	6917768	35427	4872513	1693	211221	78307	829575	86650	25920	6075090	66471	GSM5251172	SRR14265276
Adr_F_104_2	lane3_Adr_F_100_2_1	144	2	9.2	Adrenal	Ad	Adr	F	104	2	Adr-F-104	3613752	4066703	21544	2728478	1056	145623	59381	538422	63278	12868	3539688	43102	GSM5251173	SRR14265277
Adr_F_104_3	lane4_Adr_F_100_3_1	152	1.99	8.9	Adrenal	Ad	Adr	F	104	3	Adr-F-104	3222182	3697435	16319	2458946	796	200831	46835	400637	50080	13473	3163219	34265	GSM5251174	SRR14265278
Adr_F_104_4	lane5_Adr_F_100_4_1	160	1.99	8.8	Adrenal	Ad	Adr	F	104	4	Adr-F-104	5764541	6261300	32251	4697233	1450	213978	64125	635858	63818	12057	5688959	43771	GSM5251175	SRR14265279
Adr_M_002_1	lane6_Adr_M_2_1_1	129	1.95	9.2	Adrenal	Ad	Adr	M	2	1	Adr-M-2	5850008	6660375	48145	4104111	2199	86374	76416	1222493	177167	72915	5704391	60188	GSM5251176	SRR14265280
Adr_M_002_2	lane7_Adr_M_2_2_1	137	2.04	9.4	Adrenal	Ad	Adr	M	2	2	Adr-M-2	4486738	5289630	43354	2909460	2006	139619	70375	1030936	172165	64024	4376089	54799	GSM5251177	SRR14265281
Adr_M_002_3	lane7_Adr_M_2_3_1	145	1.98	9.1	Adrenal	Ad	Adr	M	2	3	Adr-M-2	4246295	4946241	40604	2833831	1922	71245	69361	954003	140536	85913	4143179	48880	GSM5251178	SRR14265282
Adr_M_002_4	lane3_Adr_M_2_4_1	153	2.02	8.9	Adrenal	Ad	Adr	M	2	4	Adr-M-2	4548261	6062117	70430	2847416	2811	69637	85899	1277531	127455	19609	4450290	47473	GSM5251179	SRR14265283
Adr_M_006_1	lane8_Adr_M_6_1_1	131	2	9.4	Adrenal	Ad	Adr	M	6	1	Adr-M-6	6436066	7758272	55661	4578909	2508	174244	105009	1227190	123935	89456	6288364	79154	GSM5251180	SRR14265284
Adr_M_006_2	lane7_Adr_M_6_2_1	139	1.99	9.3	Adrenal	Ad	Adr	M	6	2	Adr-M-6	4207899	4749416	51825	2966133	2203	95703	74235	794486	91590	66970	4101361	64754	GSM5251181	SRR14265285
Adr_M_006_3	lane5_Adr_M_6_3_1	147	2.01	9.5	Adrenal	Ad	Adr	M	6	3	Adr-M-6	4592103	5017354	38684	3456906	1589	161826	73620	643303	102918	66939	4508530	46318	GSM5251182	SRR14265286
Adr_M_006_4	lane8_Adr_M_6_4_1	155	2	9	Adrenal	Ad	Adr	M	6	4	Adr-M-6	4900977	6227058	59294	3417241	2507	196890	95250	851979	123613	78376	4768197	75827	GSM5251183	SRR14265287
Adr_M_021_1	lane1_Adr_M_21_1_1	133	1.95	8.6	Adrenal	Ad	Adr	M	21	1	Adr-M-21	5596506	6238204	19685	4772990	874	31542	72208	542773	22305	58850	5465926	75279	GSM5251184	SRR14265288
Adr_M_021_2	lane4_Adr_M_21_2_1	141	1.96	9.5	Adrenal	Ad	Adr	M	21	2	Adr-M-21	6948367	7489356	29297	5824456	1332	167254	70438	598577	54390	137145	6817295	65478	GSM5251185	SRR14265289
Adr_M_021_3	lane5_Adr_M_21_3_1	149	1.99	9.2	Adrenal	Ad	Adr	M	21	3	Adr-M-21	3916743	4215774	18671	3089294	833	109845	47281	429262	45666	137756	3833816	38135	GSM5251186	SRR14265290
Adr_M_021_4	lane1_Adr_M_21_4_1	157	1.94	9	Adrenal	Ad	Adr	M	21	4	Adr-M-21	6007506	6717236	28636	4896966	1233	199531	94890	577328	53290	61500	5846900	94132	GSM5251187	SRR14265291
Adr_M_104_1	lane2_Adr_M_100_1_1	135	1.99	9.5	Adrenal	Ad	Adr	M	104	1	Adr-M-104	4559646	5303592	22355	3447029	1153	217230	60676	624041	62689	77338	4469695	47135	GSM5251188	SRR14265292
Adr_M_104_2	lane1_Adr_M_100_2_1	143	1.99	9.4	Adrenal	Ad	Adr	M	104	2	Adr-M-104	7407930	8053233	28105	5997424	1323	352185	97446	677566	84435	54578	7218182	114868	GSM5251189	SRR14265293
Adr_M_104_3	lane4_Adr_M_100_3_1	151	2.02	8.8	Adrenal	Ad	Adr	M	104	3	Adr-M-104	7474137	8199220	37835	5915069	1841	299737	91637	898389	99627	50052	7316555	79950	GSM5251190	SRR14265294
Adr_M_104_4	lane1_Adr_M_100_4_1	159	1.99	8.9	Adrenal	Ad	Adr	M	104	4	Adr-M-104	6364269	7105482	32044	5207132	1387	199259	87465	605960	53809	79934	6199993	97279	GSM5251191	SRR14265295
Brn_F_002_1	lane2_Brn_F_2_1_1	66	2.05	9.9	Brain	Br	Brn	F	2	1	Brn-F-2	2761012	3407799	36129	2044053	1433	65695	55667	442507	38642	48907	2694507	27979	GSM5251192	SRR14265296
Brn_F_002_2	lane6_Brn_F_2_2_1	74	2.04	9.6	Brain	Br	Brn	F	2	2	Brn-F-2	4155416	4606393	32415	3315225	1363	51014	56126	572984	38475	43164	4042269	44650	GSM5251193	SRR14265297
Brn_F_002_3	lane4_Brn_F_2_3_1	82	2.04	9.1	Brain	Br	Brn	F	2	3	Brn-F-2	3528648	3919174	30889	2760031	1307	60266	49865	503333	42217	47819	3445769	32921	GSM5251194	SRR14265298
Brn_F_002_4	lane6_Brn_F_2_4_1	90	2.06	10	Brain	Br	Brn	F	2	4	Brn-F-2	3545061	3978819	34683	2750156	1504	45704	57863	533413	40698	44107	3447956	36933	GSM5251195	SRR14265299
Brn_F_006_1	lane8_Brn_F_6_1_1	68	2.05	9.1	Brain	Br	Brn	F	6	1	Brn-F-6	5099611	6015114	39084	4056823	1672	83257	81226	640223	43035	79328	4931943	74963	GSM5251196	SRR14265300
Brn_F_006_2	lane8_Msc_F_6_2_1	108	2.04	9.3	Brain	Br	Brn	F	6	2	Brn-F-6	4134539	4607510	30830	3321993	1244	43363	58709	554930	32434	40529	4015533	50507	GSM5251197	SRR14265301
Brn_F_006_3	lane7_Brn_F_6_3_1	84	2.06	9	Brain	Br	Brn	F	6	3	Brn-F-6	3719064	4077991	48597	2867311	1908	36180	71697	541145	34567	79715	3626523	37944	GSM5251198	SRR14265302
Brn_F_006_4	lane8_Brn_F_6_4_1	92	2.05	9.7	Brain	Br	Brn	F	6	4	Brn-F-6	5378997	5980697	47288	4246273	1967	57386	81460	765389	49515	64862	5223969	64857	GSM5251199	SRR14265303
Brn_F_021_1	lane2_Brn_F_21_1_1	70	2.02	9.5	Brain	Br	Brn	F	21	1	Brn-F-21	4415982	5069706	36228	3527950	1425	69053	63954	581725	36367	56135	4317671	43145	GSM5251200	SRR14265304
Brn_F_021_2	lane5_Brn_F_21_2_1	78	1.87	9.1	Brain	Br	Brn	F	21	2	Brn-F-21	5289603	6397303	39316	4258617	1616	112035	58534	646886	37682	92156	5170965	42761	GSM5251201	SRR14265305
Brn_F_021_3	lane4_Brn_F_21_3_1	86	1.9	9.1	Brain	Br	Brn	F	21	3	Brn-F-21	3346298	3879400	43665	2583531	1717	63198	66652	445706	26182	78854	3257503	36793	GSM5251202	SRR14265306
Brn_F_021_4	lane4_Brn_F_21_4_1	94	1.87	9.6	Brain	Br	Brn	F	21	4	Brn-F-21	5930367	6662147	42652	4958127	1811	46246	64223	699999	31117	36061	5799324	50131	GSM5251203	SRR14265307
Brn_F_104_1	lane3_Brn_F_100_1_1	72	2.04	9.3	Brain	Br	Brn	F	104	1	Brn-F-104	2172847	2476272	15249	1782185	668	21501	22284	294667	11041	10394	2132073	14858	GSM5251204	SRR14265308
Brn_F_104_2	lane7_Brn_F_100_2_1	80	1.91	9.2	Brain	Br	Brn	F	104	2	Brn-F-104	3799352	4327576	64069	2883735	2465	63061	96403	550394	33923	61614	3700526	43688	GSM5251205	SRR14265309
Brn_F_104_3	lane6_Brn_F_100_3_1	88	1.96	9.7	Brain	Br	Brn	F	104	3	Brn-F-104	5230787	5651671	35471	4258441	1532	66046	69129	656314	40102	46844	5101353	56908	GSM5251206	SRR14265310
Brn_F_104_4	lane5_Brn_F_100_4_1	96	1.97	9.6	Brain	Br	Brn	F	104	4	Brn-F-104	8073603	10171422	491226	1164538	27396	143823	1032456	3971449	744549	384141	7865317	114025	GSM5251207	SRR14265311
Brn_M_002_1	lane1_Brn_M_2_1_1	65	2.06	10	Brain	Br	Brn	M	2	1	Brn-M-2	2274091	2644964	22703	1742829	993	49501	45728	314779	34219	25512	2195165	37827	GSM5251208	SRR14265312
Brn_M_002_2	lane1_Brn_M_2_2_1	73	2.04	9.9	Brain	Br	Brn	M	2	2	Brn-M-2	3460614	3837464	30750	2628208	1222	94080	68664	451749	51877	73683	3335124	60381	GSM5251209	SRR14265313
Brn_M_002_3	lane7_Brn_M_2_3_1	81	2.06	9.6	Brain	Br	Brn	M	2	3	Brn-M-2	2459211	2809048	27207	1826961	1206	35742	42683	409281	40970	50420	2397256	24741	GSM5251210	SRR14265314
Brn_M_002_4	lane3_Brn_M_2_4_1	89	2.03	9.9	Brain	Br	Brn	M	2	4	Brn-M-2	2152886	2508362	38940	1625754	1468	27958	55056	336649	26317	19736	2103356	21008	GSM5251211	SRR14265315
Brn_M_006_1	lane8_Brn_M_6_1_1	67	2.05	9.6	Brain	Br	Brn	M	6	1	Brn-M-6	4850860	5670608	36375	3805451	1501	87718	74856	601808	43717	131245	4694656	68189	GSM5251212	SRR14265316
Brn_M_006_2	lane8_Brn_M_6_2_1	75	1.93	9.3	Brain	Br	Brn	M	6	2	Brn-M-6	4897491	5661683	39247	3868768	1717	52915	62133	714502	39884	65398	4769287	52927	GSM5251213	SRR14265317
Brn_M_006_3	lane3_Brn_M_6_3_1	83	2.08	9.1	Brain	Br	Brn	M	6	3	Brn-M-6	9415750	10794663	93605	6570833	4952	101421	247153	2117922	97791	105057	9232879	77016	GSM5251214	SRR14265318
Brn_M_006_4	lane7_Brn_M_6_4_1	91	2.03	9.4	Brain	Br	Brn	M	6	4	Brn-M-6	3030237	3351936	24358	2407294	959	36433	37599	437430	27001	33441	2961791	25722	GSM5251215	SRR14265319
Brn_M_021_1	lane5_Brn_M_21_1_1	69	2.03	9.6	Brain	Br	Brn	M	21	1	Brn-M-21	4095321	4409322	29185	3271606	1287	56583	57073	550549	34528	62766	4013662	31744	GSM5251216	SRR14265320
Brn_M_021_2	lane3_Brn_M_21_2_1	77	1.98	8.9	Brain	Br	Brn	M	21	2	Brn-M-21	4591697	5319210	36801	3699507	1732	43127	64512	632500	33364	42263	4496707	37891	GSM5251217	SRR14265321
Brn_M_021_3	lane2_Brn_M_21_3_1	85	2.05	9.2	Brain	Br	Brn	M	21	3	Brn-M-21	3495892	4020069	28874	2767837	1241	54873	52031	478907	30472	51285	3421863	30372	GSM5251218	SRR14265322
Brn_M_021_4	lane4_Brn_M_21_4_1	93	2.04	9.2	Brain	Br	Brn	M	21	4	Brn-M-21	5937402	6489412	42798	4777951	1776	87576	78857	741158	48743	102076	5791620	56467	GSM5251219	SRR14265323
Brn_M_104_1	lane6_Brn_M_100_1_1	71	1.93	9.5	Brain	Br	Brn	M	104	1	Brn-M-104	5115143	5689156	37340	4151315	1470	51804	65495	621537	40512	86224	4963904	59446	GSM5251220	SRR14265324
Brn_M_104_2	lane2_Brn_M_100_2_1	79	1.92	9.3	Brain	Br	Brn	M	104	2	Brn-M-104	3690249	4268520	26058	2967320	1083	65183	49822	478184	36013	35314	3611959	31272	GSM5251221	SRR14265325
Brn_M_104_3	lane1_Brn_M_100_3_1	87	1.96	9.5	Brain	Br	Brn	M	104	3	Brn-M-104	4739826	5301027	36299	3761065	1569	77717	79204	602505	50652	55155	4589501	75660	GSM5251222	SRR14265326
Brn_M_104_4	lane5_Brn_M_100_4_1	95	2.05	9.3	Brain	Br	Brn	M	104	4	Brn-M-104	3841027	4172736	27706	3084909	1132	63041	52613	484219	39121	54382	3758954	33904	GSM5251223	SRR14265327
Hrt_F_002_1	lane3_Hrt_F_2_1_1	162	1.96	9.4	Heart	He	Hrt	F	2	1	Hrt-F-2	4001639	4660618	43511	3255248	1381	9909	46716	569607	40443	11911	3943151	22913	GSM5251224	SRR14265328
Hrt_F_002_2	lane2_Hrt_F_2_2_1	170	1.91	9.5	Heart	He	Hrt	F	2	2	Hrt-F-2	3617402	4345074	58211	2744112	1755	16547	68932	607877	65024	21455	3548650	33489	GSM5251225	SRR14265329
Hrt_F_002_3	lane5_Hrt_F_2_3_1	178	1.84	9.3	Heart	He	Hrt	F	2	3	Hrt-F-2	3084842	3402660	50313	2337243	1531	13598	56580	507007	74388	19145	3029514	25037	GSM5251226	SRR14265330
Hrt_F_002_4	lane1_Hrt_F_2_4_1	186	1.91	9.1	Heart	He	Hrt	F	2	4	Hrt-F-2	3883907	4582120	55771	2893566	1763	16444	76451	675899	85730	21099	3776207	57184	GSM5251227	SRR14265331
Hrt_F_006_1	lane5_Hrt_F_6_1_1	164	2.04	9.3	Heart	He	Hrt	F	6	1	Hrt-F-6	1839275	2021111	31758	1479441	833	4655	28235	243364	27027	10789	1808892	13173	GSM5251228	SRR14265332
Hrt_F_006_2	lane2_Hrt_F_6_2_1	172	2.04	9.3	Heart	He	Hrt	F	6	2	Hrt-F-6	3045779	3766112	48852	2337278	1354	9319	56639	471903	56919	29904	2982104	33611	GSM5251229	SRR14265333
Hrt_F_006_3	lane6_Hrt_F_6_3_1	180	1.95	9	Heart	He	Hrt	F	6	3	Hrt-F-6	6088777	6775280	78063	4673460	2575	13687	98652	1046110	95376	20775	5966184	60079	GSM5251230	SRR14265334
Hrt_F_006_4	lane7_Hrt_F_6_4_1	188	2.06	8.9	Heart	He	Hrt	F	6	4	Hrt-F-6	3901204	4666958	72532	2551192	2553	13085	92209	994203	102084	18050	3806037	55296	GSM5251231	SRR14265335
Hrt_F_021_1	lane2_Hrt_F_21_1_1	166	2.01	9	Heart	He	Hrt	F	21	1	Hrt-F-21	4457372	5701188	83742	2848789	2574	13867	104138	1156064	156625	27944	4350072	63629	GSM5251232	SRR14265336
Hrt_F_021_2	lane7_Hrt_F_21_2_1	174	2.01	8.8	Heart	He	Hrt	F	21	2	Hrt-F-21	4229132	5136551	85322	2700336	2612	12877	96511	1081879	168606	24625	4126884	56364	GSM5251233	SRR14265337
Hrt_F_021_3	lane1_Hrt_F_21_3_1	182	2	8.8	Heart	He	Hrt	F	21	3	Hrt-F-21	3996171	4805430	63851	2816800	1990	11211	96445	809317	106346	19440	3876576	70771	GSM5251234	SRR14265338
Hrt_F_021_4	lane8_Hrt_F_21_4_1	190	1.98	8.7	Heart	He	Hrt	F	21	4	Hrt-F-21	4631192	6080314	94504	3029223	2825	13879	116766	1099917	152848	31684	4483974	89546	GSM5251235	SRR14265339
Hrt_F_104_1	lane8_Hrt_F_100_1_1	168	2.01	9.1	Heart	He	Hrt	F	104	1	Hrt-F-104	4345320	5635231	73892	2981999	2395	13330	107437	884514	110493	87193	4189442	84067	GSM5251236	SRR14265340
Hrt_F_104_2	lane2_Hrt_F_100_2_1	176	2.02	8.7	Heart	He	Hrt	F	104	2	Hrt-F-104	4228971	5146149	48623	3150333	1589	10749	89871	739746	79108	50150	4123178	58802	GSM5251237	SRR14265341
Hrt_F_104_3	lane3_Hrt_F_100_3_1	184	2.04	8.8	Heart	He	Hrt	F	104	3	Hrt-F-104	4327592	4976946	40060	3419763	1221	8398	72952	654768	38274	48392	4230356	43764	GSM5251238	SRR14265342
Hrt_F_104_4	lane4_Hrt_F_100_4_1	192	2	8.7	Heart	He	Hrt	F	104	4	Hrt-F-104	5235553	6030346	121209	3511535	3277	16973	132228	1200675	90974	88650	5079134	70032	GSM5251239	SRR14265343
Hrt_M_002_1	lane8_Hrt_M_2_1_1	161	2.06	9.7	Heart	He	Hrt	M	2	1	Hrt-M-2	4695165	5362180	73096	3462099	2389	17720	81760	898044	92188	21627	4590992	46242	GSM5251240	SRR14265344
Hrt_M_002_2	lane6_Hrt_M_2_2_1	169	2.04	9.5	Heart	He	Hrt	M	2	2	Hrt-M-2	4361984	4994616	66929	3232588	2240	15014	70956	821204	94863	18044	4272207	40146	GSM5251241	SRR14265345
Hrt_M_002_3	lane5_Hrt_M_2_3_1	177	2.07	9.5	Heart	He	Hrt	M	2	3	Hrt-M-2	3446777	3837306	53117	2603448	1643	13426	52921	618601	66383	15177	3393628	22061	GSM5251242	SRR14265346
Hrt_M_002_4	lane6_Hrt_M_2_4_1	185	2.06	9.2	Heart	He	Hrt	M	2	4	Hrt-M-2	5129149	5771681	92200	3788343	2923	18541	89805	982173	84988	22714	5028655	47462	GSM5251243	SRR14265347
Hrt_M_006_1	lane7_Hrt_M_6_1_1	163	2.05	9	Heart	He	Hrt	M	6	1	Hrt-M-6	4639291	5069500	65252	3706515	1907	11117	78967	650004	66472	20132	4545190	38925	GSM5251244	SRR14265348
Hrt_M_006_2	lane4_Hrt_M_6_2_1	171	2.04	9.1	Heart	He	Hrt	M	6	2	Hrt-M-6	4902817	5551470	60659	3989853	1882	9127	64534	674234	48938	13222	4814615	40368	GSM5251245	SRR14265349
Hrt_M_006_3	lane4_Hrt_M_6_3_1	179	2.03	9.2	Heart	He	Hrt	M	6	3	Hrt-M-6	4616097	5112506	52946	3734466	1616	10351	70878	622193	57822	19444	4521705	46381	GSM5251246	SRR14265350
Hrt_M_006_4	lane1_Hrt_M_6_4_1	187	2.04	8.7	Heart	He	Hrt	M	6	4	Hrt-M-6	4697864	5372437	83531	3524231	2343	13193	97234	785064	76232	44954	4564661	71082	GSM5251247	SRR14265351
Hrt_M_021_1	lane1_Hrt_M_21_1_1	165	2.02	8.7	Heart	He	Hrt	M	21	1	Hrt-M-21	4635503	5298234	63727	3154429	2015	13549	129015	951265	170298	46264	4462386	104941	GSM5251248	SRR14265352
Hrt_M_021_2	lane3_Hrt_M_21_2_1	173	2.02	8.7	Heart	He	Hrt	M	21	2	Hrt-M-21	3754766	4433841	71185	2659905	2017	8409	77852	794097	82550	17052	3675984	41699	GSM5251249	SRR14265353
Hrt_M_021_3	lane6_Hrt_M_21_3_1	181	2	8.7	Heart	He	Hrt	M	21	3	Hrt-M-21	4636124	5315382	72756	3287335	2177	10277	100571	966437	111210	21744	4518470	63617	GSM5251250	SRR14265354
Hrt_M_021_4	lane7_Hrt_M_21_4_1	189	2.02	8.5	Heart	He	Hrt	M	21	4	Hrt-M-21	3276534	3806879	61428	2292434	1787	8723	79817	678321	79407	28721	3188688	45896	GSM5251251	SRR14265355
Hrt_M_104_1	lane5_Hrt_M_100_1_1	167	1.94	8.7	Heart	He	Hrt	M	104	1	Hrt-M-104	3750972	4294669	181264	2570304	4419	13724	150997	639967	66561	65562	3641373	58174	GSM5251252	SRR14265356
Hrt_M_104_2	lane4_Hrt_M_100_2_1	175	2.03	8.9	Heart	He	Hrt	M	104	2	Hrt-M-104	2907554	3486439	44416	2146292	1426	8995	69834	508208	51302	36096	2828958	40985	GSM5251253	SRR14265357
Hrt_M_104_3	lane3_Hrt_M_100_3_1	183	1.96	8.6	Heart	He	Hrt	M	104	3	Hrt-M-104	3605471	4201295	116295	2571497	2913	11509	112122	654381	43710	44090	3502481	48954	GSM5251254	SRR14265358
Hrt_M_104_4	lane8_Hrt_M_100_4_1	191	2.01	8.6	Heart	He	Hrt	M	104	4	Hrt-M-104	5454066	6611809	93085	3859712	2877	12986	113773	1134166	92033	70632	5298688	74802	GSM5251255	SRR14265359
Kdn_F_002_1	lane8_Kdn_F_2_1_1	194	2.04	9.3	Kidney	Ki	Kdn	F	2	1	Kdn-F-2	7305621	8222467	50940	6296879	2285	16661	82941	616329	68867	107154	7180338	63565	GSM5251256	SRR14265360
Kdn_F_002_2	lane2_Kdn_F_2_2_1	202	2.01	8	Kidney	Ki	Kdn	F	2	2	Kdn-F-2	6640111	7828863	73989	4272806	2859	22074	174460	1372921	111751	547109	6457922	62142	GSM5251257	SRR14265361
Kdn_F_002_3	lane2_Kdn_F_2_3_1	210	2.02	8.5	Kidney	Ki	Kdn	F	2	3	Kdn-F-2	5678408	6747132	53524	4431793	2372	18656	68256	923887	110994	31459	5606774	37467	GSM5251258	SRR14265362
Kdn_F_002_4	lane6_Kdn_F_2_4_1	218	2	8.8	Kidney	Ki	Kdn	F	2	4	Kdn-F-2	6523032	7175493	47962	5335308	2162	14675	71042	841827	122479	30688	6419851	56889	GSM5251259	SRR14265363
Kdn_F_006_1	lane6_Kdn_F_6_1_1	196	2.04	8.8	Kidney	Ki	Kdn	F	6	1	Kdn-F-6	6862483	7600319	51009	5547836	2037	13727	84435	949223	57847	93717	6720185	62652	GSM5251260	SRR14265364
Kdn_F_006_2	lane8_Kdn_F_6_2_1	204	1.95	8.5	Kidney	Ki	Kdn	F	6	2	Kdn-F-6	6732885	7471832	48189	5378821	2140	15142	89790	936896	100027	91042	6591919	70838	GSM5251261	SRR14265365
Kdn_F_006_3	lane3_Kdn_F_6_3_1	212	1.97	8.5	Kidney	Ki	Kdn	F	6	3	Kdn-F-6	4877469	5548147	37142	3871383	1628	10212	53031	767554	66514	38463	4804615	31542	GSM5251262	SRR14265366
Kdn_F_006_4	lane6_Kdn_F_6_4_1	220	2.05	9	Kidney	Ki	Kdn	F	6	4	Kdn-F-6	6507453	7019471	49695	5380203	2152	13992	80773	768199	69302	89451	6404407	53686	GSM5251263	SRR14265367
Kdn_F_021_1	lane5_Kdn_F_21_1_1	198	1.96	8.6	Kidney	Ki	Kdn	F	21	1	Kdn-F-21	6170901	6662524	39529	4891115	1736	17125	71379	785214	104726	214171	6060162	45906	GSM5251264	SRR14265368
Kdn_F_021_2	lane8_Kdn_F_21_2_1	206	2.03	8.3	Kidney	Ki	Kdn	F	21	2	Kdn-F-21	7508310	8525495	52408	6267251	2228	16172	88427	891692	74417	47063	7386788	68652	GSM5251265	SRR14265369
Kdn_F_021_3	lane5_Kdn_F_21_3_1	214	2.02	8.5	Kidney	Ki	Kdn	F	21	3	Kdn-F-21	3563971	3855026	24063	2930118	1061	11251	48901	415680	63781	41113	3515283	28003	GSM5251266	SRR14265370
Kdn_F_021_4	lane7_Kdn_F_21_4_1	222	1.99	8.7	Kidney	Ki	Kdn	F	21	4	Kdn-F-21	4474378	4977954	30394	3579686	1331	11721	52594	663114	83017	18467	4416026	34054	GSM5251267	SRR14265371
Kdn_F_104_1	lane2_Kdn_F_100_1_1	200	1.94	8.8	Kidney	Ki	Kdn	F	104	1	Kdn-F-104	4681092	5447814	33071	3732760	1474	14045	60510	702629	79406	22866	4623435	34331	GSM5251268	SRR14265372
Kdn_F_104_2	lane4_Kdn_F_100_2_1	208	1.99	8.2	Kidney	Ki	Kdn	F	104	2	Kdn-F-104	6155094	6665759	37853	5197158	1621	14638	70797	683186	72190	28421	6071938	49230	GSM5251269	SRR14265373
Kdn_F_104_3	lane6_Kdn_F_100_3_1	216	1.98	8.6	Kidney	Ki	Kdn	F	104	3	Kdn-F-104	5574729	6145903	39697	4461699	1752	12749	63901	812527	92443	40065	5485945	49896	GSM5251270	SRR14265374
Kdn_F_104_4	lane5_Kdn_F_100_4_1	224	1.98	9.6	Kidney	Ki	Kdn	F	104	4	Kdn-F-104	5817044	6287911	32887	4853867	1427	13118	57447	724920	86478	14406	5759955	32494	GSM5251271	SRR14265375
Kdn_M_002_1	lane3_Kdn_M_2_1_1	193	2.01	8.6	Kidney	Ki	Kdn	M	2	1	Kdn-M-2	6167080	7302992	66927	4626341	2855	16573	69048	1160456	111054	75465	6079335	38361	GSM5251272	SRR14265376
Kdn_M_002_2	lane7_Kdn_M_2_2_1	201	1.99	9	Kidney	Ki	Kdn	M	2	2	Kdn-M-2	5858782	6499659	44691	4710106	2091	13945	61659	810576	117661	58217	5779611	39836	GSM5251273	SRR14265377
Kdn_M_002_3	lane1_Kdn_M_2_3_1	209	1.99	9.3	Kidney	Ki	Kdn	M	2	3	Kdn-M-2	8584251	9445841	65944	6962767	2848	21963	117265	1041639	186891	74374	8399814	110560	GSM5251274	SRR14265378
Kdn_M_002_4	lane7_Kdn_M_2_4_1	217	1.96	9.3	Kidney	Ki	Kdn	M	2	4	Kdn-M-2	5899383	6548999	47920	4886374	2105	16027	66616	699397	95163	47694	5821018	38087	GSM5251275	SRR14265379
Kdn_M_006_1	lane4_Kdn_M_6_1_1	195	2.03	8.8	Kidney	Ki	Kdn	M	6	1	Kdn-M-6	6567202	7350426	49252	5395381	2071	12567	74907	847855	65171	68138	6466495	51860	GSM5251276	SRR14265380
Kdn_M_006_2	lane4_Kdn_M_6_2_1	203	2.04	8.5	Kidney	Ki	Kdn	M	6	2	Kdn-M-6	5730309	6298728	52914	4527151	2122	14915	77044	794042	93337	122538	5633920	46246	GSM5251277	SRR14265381
Kdn_M_006_3	lane5_Kdn_M_6_3_1	211	2.05	8.2	Kidney	Ki	Kdn	M	6	3	Kdn-M-6	4856411	5181974	24377	4267931	1022	9395	43675	415027	32142	39128	4806411	23714	GSM5251278	SRR14265382
Kdn_M_006_4	lane1_Kdn_M_6_4_1	219	2.03	9	Kidney	Ki	Kdn	M	6	4	Kdn-M-6	5268556	5983886	37635	4363689	1586	13483	79615	602031	70003	32664	5157775	67850	GSM5251279	SRR14265383
Kdn_M_021_1	lane7_Kdn_M_21_1_1	197	2.05	8.5	Kidney	Ki	Kdn	M	21	1	Kdn-M-21	6667952	7121549	28569	5773234	1335	12043	60929	610594	53180	85254	6579955	42814	GSM5251280	SRR14265384
Kdn_M_021_2	lane1_Kdn_M_21_2_1	205	1.88	8.3	Kidney	Ki	Kdn	M	21	2	Kdn-M-21	5017153	5525851	27681	4235736	1239	11705	70707	526130	50352	29831	4915142	63772	GSM5251281	SRR14265385
Kdn_M_021_3	lane1_Kdn_M_21_3_1	213	2.04	8.1	Kidney	Ki	Kdn	M	21	3	Kdn-M-21	2297095	2603455	16102	1919729	699	6864	38905	234176	25915	21011	2243725	33694	GSM5251282	SRR14265386
Kdn_M_021_4	lane3_Kdn_M_21_4_1	221	1.89	8.9	Kidney	Ki	Kdn	M	21	4	Kdn-M-21	3906893	4347623	26986	3215322	1161	10942	53012	483353	52828	29744	3846798	33545	GSM5251283	SRR14265387
Kdn_M_104_1	lane8_Kdn_M_100_1_1	199	1.92	8.3	Kidney	Ki	Kdn	M	104	1	Kdn-M-104	3837815	4471207	24217	3212905	1020	10726	49231	410615	49237	39692	3766492	40172	GSM5251284	SRR14265388
Kdn_M_104_2	lane3_Kdn_M_100_2_1	207	/	/	Kidney	Ki	Kdn	M	104	2	Kdn-M-104	4077110	4725220	31601	3341020	1408	9100	38441	556927	59480	15369	4031475	23764	GSM5251285	SRR14265389
Kdn_M_104_3	lane2_Kdn_M_100_3_1	215	/	/	Kidney	Ki	Kdn	M	104	3	Kdn-M-104	4140469	5071204	32066	3105504	1406	12145	48246	665891	104138	139831	4064518	31242	GSM5251286	SRR14265390
Kdn_M_104_4	lane4_Kdn_M_100_4_1	223	1.95	8.9	Kidney	Ki	Kdn	M	104	4	Kdn-M-104	4218158	4819804	29554	3405567	1358	13740	56055	572531	66521	36895	4154756	35937	GSM5251287	SRR14265391
Lng_F_002_1	lane1_Lng_F_2_1_1	290	2.05	9.5	Lung	Lu	Lng	F	2	1	Lng-F-2	8097693	9016357	52779	6821529	2217	39099	99130	849972	72834	75349	7908708	84784	GSM5251288	SRR14265392
Lng_F_002_2	lane7_Lng_F_2_2_1	298	2.04	9.3	Lung	Lu	Lng	F	2	2	Lng-F-2	4822097	5299969	30107	4101055	1374	20026	45470	519307	55180	22350	4749531	27228	GSM5251289	SRR14265393
Lng_F_002_3	lane5_Lng_F_2_3_1	306	2.05	9.7	Lung	Lu	Lng	F	2	3	Lng-F-2	4132154	4405657	23719	3504599	933	17874	37554	440276	48543	34366	4070799	24290	GSM5251290	SRR14265394
Lng_F_002_4	lane7_Lng_F_2_4_1	314	2.05	9.6	Lung	Lu	Lng	F	2	4	Lng-F-2	5558254	6018649	37727	4777661	1501	20119	57080	557926	46489	25741	5470863	34010	GSM5251291	SRR14265395
Lng_F_006_1	lane6_Lng_F_6_1_1	292	2.05	9.3	Lung	Lu	Lng	F	6	1	Lng-F-6	6487594	7017061	52340	5495104	1828	14630	76444	699963	57006	46244	6379681	44035	GSM5251292	SRR14265396
Lng_F_006_2	lane5_Lng_F_6_2_1	300	2.01	9.4	Lung	Lu	Lng	F	6	2	Lng-F-6	3931779	4230601	28155	3250597	1080	14155	42862	457220	54959	63241	3875777	19510	GSM5251293	SRR14265397
Lng_F_006_3	lane4_Lng_F_6_3_1	308	2.03	9.3	Lung	Lu	Lng	F	6	3	Lng-F-6	4146700	4639157	28414	3442381	1158	14790	42574	519504	48108	25719	4085262	24052	GSM5251294	SRR14265398
Lng_F_006_4	lane6_Lng_F_6_4_1	316	2.06	9.5	Lung	Lu	Lng	F	6	4	Lng-F-6	6074027	6489650	42320	5100845	1597	14349	66815	701627	61494	44164	5977781	40816	GSM5251295	SRR14265399
Lng_F_021_1	lane4_Lng_F_21_1_1	294	2.03	9.4	Lung	Lu	Lng	F	21	1	Lng-F-21	7334144	7876863	37727	6348047	1530	18364	70994	698831	42749	70611	7218361	45291	GSM5251296	SRR14265400
Lng_F_021_2	lane2_Lng_F_21_2_1	302	2.02	9.4	Lung	Lu	Lng	F	21	2	Lng-F-21	5794461	6409164	33854	4989623	1419	19009	55655	563603	52744	53254	5731414	25300	GSM5251297	SRR14265401
Lng_F_021_3	lane8_Lng_F_21_3_1	310	1.97	9.4	Lung	Lu	Lng	F	21	3	Lng-F-21	5408715	6051883	29256	4660996	1229	19369	64292	517318	46167	29287	5322244	40801	GSM5251298	SRR14265402
Lng_F_021_4	lane3_Lng_F_21_4_1	318	2.04	9.8	Lung	Lu	Lng	F	21	4	Lng-F-21	3895285	4200231	19676	3413975	781	11585	36481	348898	29425	15432	3847478	19032	GSM5251299	SRR14265403
Lng_F_104_1	lane8_Lng_F_100_1_1	296	2.03	9.5	Lung	Lu	Lng	F	104	1	Lng-F-104	4669873	5286025	47231	3849694	2298	17425	61391	584814	54199	19300	4592427	33521	GSM5251300	SRR14265404
Lng_F_104_2	lane3_Lng_F_100_2_1	304	2	9.3	Lung	Lu	Lng	F	104	2	Lng-F-104	5501733	6168401	41802	4608342	1883	17655	56426	689080	52542	10475	5436671	23528	GSM5251301	SRR14265405
Lng_F_104_3	lane7_Lng_F_100_3_1	312	1.92	9.6	Lung	Lu	Lng	F	104	3	Lng-F-104	6646675	7126002	35772	5711261	1638	18093	67588	693941	61816	24165	6564018	32401	GSM5251302	SRR14265406
Lng_F_104_4	lane1_Lng_F_100_4_1	320	1.92	9.6	Lung	Lu	Lng	F	104	4	Lng-F-104	5322547	5864113	38642	4423116	1784	19425	77842	633314	56409	17512	5215091	54503	GSM5251303	SRR14265407
Lng_M_002_1	lane8_Lng_M_2_1_1	289	2.02	9.6	Lung	Lu	Lng	M	2	1	Lng-M-2	7103880	8088214	50875	5956391	2183	36933	81139	794511	85629	41832	6965013	54387	GSM5251304	SRR14265408
Lng_M_002_2	lane3_Lng_M_2_2_1	297	2.04	9.7	Lung	Lu	Lng	M	2	2	Lng-M-2	5530601	6149600	38497	4739634	1706	15359	48988	606244	38663	17949	5459587	23561	GSM5251305	SRR14265409
Lng_M_002_3	lane5_Lng_M_2_3_1	305	2.05	9.7	Lung	Lu	Lng	M	2	3	Lng-M-2	4553691	4862504	27680	3879963	1135	17829	39925	481051	41877	43341	4489178	20890	GSM5251306	SRR14265410
Lng_M_002_4	lane1_Lng_M_2_4_1	313	2.04	9.5	Lung	Lu	Lng	M	2	4	Lng-M-2	6517180	7143824	64503	5344403	2554	30077	112639	754784	100974	27956	6359541	79290	GSM5251307	SRR14265411
Lng_M_006_1	lane2_Lng_M_6_1_1	291	1.94	9.4	Lung	Lu	Lng	M	6	1	Lng-M-6	5217196	5868231	32186	4306647	1349	19546	51932	682016	69465	29939	5153128	24116	GSM5251308	SRR14265412
Lng_M_006_2	lane4_Lng_M_6_2_1	299	2.06	9.4	Lung	Lu	Lng	M	6	2	Lng-M-6	5618381	6076380	40184	4740752	1674	17866	58793	639038	67022	23048	5539953	30004	GSM5251309	SRR14265413
Lng_M_006_3	lane8_Lng_M_6_3_1	307	2.04	9.5	Lung	Lu	Lng	M	6	3	Lng-M-6	6959463	7574204	39448	5961932	1564	16908	65919	737995	60525	31607	6847339	43565	GSM5251310	SRR14265414
Lng_M_006_4	lane5_Lng_M_6_4_1	315	2.06	9.5	Lung	Lu	Lng	M	6	4	Lng-M-6	4512834	4827741	26630	3900194	995	12123	42005	439137	33600	37948	4457960	20202	GSM5251311	SRR14265415
Lng_M_021_1	lane3_Lng_M_21_1_1	293	1.93	9.2	Lung	Lu	Lng	M	21	1	Lng-M-21	6151207	6731612	31770	5160093	1447	14335	53535	747539	46559	70255	6056666	25674	GSM5251312	SRR14265416
Lng_M_021_2	lane1_Lng_M_21_2_1	301	2.04	9.1	Lung	Lu	Lng	M	21	2	Lng-M-21	5181170	5741540	28137	4392375	1197	17180	82249	544140	32750	31386	5074047	51756	GSM5251313	SRR14265417
Lng_M_021_3	lane6_Lng_M_21_3_1	309	2.02	9.2	Lung	Lu	Lng	M	21	3	Lng-M-21	3769121	4172660	26946	3150107	1070	9814	42386	417966	36050	56120	3694063	28662	GSM5251314	SRR14265418
Lng_M_021_4	lane2_Lng_M_21_4_1	317	1.98	9.6	Lung	Lu	Lng	M	21	4	Lng-M-21	3965451	4540843	24835	3242471	986	14335	41545	484865	43121	94042	3905883	19251	GSM5251315	SRR14265419
Lng_M_104_1	lane6_Lng_M_100_1_1	295	1.97	9.3	Lung	Lu	Lng	M	104	1	Lng-M-104	6367977	6899536	44728	5422815	1974	16162	87010	646153	59160	35439	6253344	54536	GSM5251316	SRR14265420
Lng_M_104_2	lane2_Lng_M_100_2_1	303	1.97	9.4	Lung	Lu	Lng	M	104	2	Lng-M-104	3968944	4516475	25645	3174123	1108	19838	51593	592658	60909	20218	3915074	22852	GSM5251317	SRR14265421
Lng_M_104_3	lane4_Lng_M_100_3_1	311	2.05	9.2	Lung	Lu	Lng	M	104	3	Lng-M-104	7276341	7887080	44469	6295820	1950	16430	74112	737752	40431	26383	7174791	38994	GSM5251318	SRR14265422
Lng_M_104_4	lane7_Lng_M_100_4_1	319	1.97	9.5	Lung	Lu	Lng	M	104	4	Lng-M-104	4560906	5008451	34531	3731878	1509	16330	53823	605211	70232	21960	4496531	25432	GSM5251319	SRR14265423
Lvr_F_002_1	lane7_Lvr_F_2_1_1	34	2.02	9.2	Liver	Li	Lvr	F	2	1	Lvr-F-2	1658369	1950038	23837	1154707	928	29134	35271	342661	44159	7134	1607963	20538	GSM5251320	SRR14265424
Lvr_F_002_2	lane6_Lvr_F_2_2_1	42	2.02	9.3	Liver	Li	Lvr	F	2	2	Lvr-F-2	1535374	1788084	21556	1075249	789	21261	31304	312627	42441	6878	1478653	23269	GSM5251321	SRR14265425
Lvr_F_002_3	lane6_Lvr_F_2_3_1	50	2.05	9	Liver	Li	Lvr	F	2	3	Lvr-F-2	2137589	2440461	27907	1492124	1066	27167	39873	473498	38265	8737	2066050	28952	GSM5251322	SRR14265426
Lvr_F_002_4	lane1_Lvr_F_2_4_1	58	1.98	8.8	Liver	Li	Lvr	F	2	4	Lvr-F-2	1695520	2043781	25083	1164695	968	30967	43696	347885	40727	9520	1634453	31979	GSM5251323	SRR14265427
Lvr_F_006_1	lane3_Lvr_F_6_1_1	36	1.94	9.5	Liver	Li	Lvr	F	6	1	Lvr-F-6	453764	548898	8179	315551	281	2774	9827	91272	15388	2687	440454	7805	GSM5251324	SRR14265428
Lvr_F_006_2	lane1_Lvr_F_6_2_1	44	2.02	9.3	Liver	Li	Lvr	F	6	2	Lvr-F-6	864746	1066184	12613	612106	454	5599	20043	167040	26122	4020	836343	16749	GSM5251325	SRR14265429
Lvr_F_006_3	lane5_Lvr_F_6_3_1	52	2.05	9.2	Liver	Li	Lvr	F	6	3	Lvr-F-6	1205591	1382618	17844	852789	639	8702	24267	244582	32619	8502	1175855	15647	GSM5251326	SRR14265430
Lvr_F_006_4	lane4_Lvr_F_6_4_1	60	2.01	9.6	Liver	Li	Lvr	F	6	4	Lvr-F-6	1440506	1666293	21675	1034426	779	8697	31716	282051	36538	7111	1407143	17513	GSM5251327	SRR14265431
Lvr_F_021_1	lane2_Lvr_F_21_1_1	38	1.91	8.7	Liver	Li	Lvr	F	21	1	Lvr-F-21	902873	1183627	13460	614572	510	5618	21619	188667	26082	20997	879345	11348	GSM5251328	SRR14265432
Lvr_F_021_2	lane8_Lvr_F_21_2_1	46	2.03	8.8	Liver	Li	Lvr	F	21	2	Lvr-F-21	1690379	2001461	18177	1304083	679	6344	25084	283777	23040	9882	1649733	19313	GSM5251329	SRR14265433
Lvr_F_021_3	lane4_Lvr_F_21_3_1	54	2.04	8.8	Liver	Li	Lvr	F	21	3	Lvr-F-21	1977816	2254434	25966	1429609	974	10398	32059	397006	43847	16502	1931092	21455	GSM5251330	SRR14265434
Lvr_F_021_4	lane1_Lvr_F_21_4_1	62	1.87	9.4	Liver	Li	Lvr	F	21	4	Lvr-F-21	1580913	1841444	19925	1155668	783	8447	37770	276309	39693	12693	1529988	29625	GSM5251331	SRR14265435
Lvr_F_104_1	lane8_Lvr_F_100_1_1	40	2.04	9.3	Liver	Li	Lvr	F	104	1	Lvr-F-104	2012729	2536087	31883	1448340	1223	11707	35836	382569	63792	13467	1967228	23912	GSM5251332	SRR14265436
Lvr_F_104_2	lane5_Lvr_F_100_2_1	48	1.88	9	Liver	Li	Lvr	F	104	2	Lvr-F-104	1379159	1593879	23803	953878	789	7133	21982	309403	42262	5554	1351043	14355	GSM5251333	SRR14265437
Lvr_F_104_3	lane5_Lvr_F_100_3_1	56	2.03	9.2	Liver	Li	Lvr	F	104	3	Lvr-F-104	988532	1111008	11657	678243	391	5709	15774	234274	26460	4460	965858	11564	GSM5251334	SRR14265438
Lvr_F_104_4	lane3_Lvr_F_100_4_1	64	2.02	9.3	Liver	Li	Lvr	F	104	4	Lvr-F-104	1532610	1789574	17869	1118522	655	6429	19536	311824	37923	6962	1503176	12890	GSM5251335	SRR14265439
Lvr_M_002_1	lane7_Lvr_M_2_1_1	33	2	9	Liver	Li	Lvr	M	2	1	Lvr-M-2	1563501	1727984	18529	1169365	758	26749	29384	265258	30099	5355	1506824	18004	GSM5251336	SRR14265440
Lvr_M_002_2	lane6_Lvr_M_2_2_1	41	2.03	9.2	Liver	Li	Lvr	M	2	2	Lvr-M-2	2546157	2919496	28548	1692153	1099	56749	42361	607947	77078	6195	2454530	34027	GSM5251337	SRR14265441
Lvr_M_002_3	lane8_Lvr_M_2_3_1	49	2.04	8.8	Liver	Li	Lvr	M	2	3	Lvr-M-2	1991512	2381336	29744	1298929	1044	40137	41178	483644	57417	8931	1911012	30488	GSM5251338	SRR14265442
Lvr_M_002_4	lane6_Lvr_M_2_4_1	57	2.03	9.8	Liver	Li	Lvr	M	2	4	Lvr-M-2	1492746	1799603	18746	1114671	728	29714	24812	255888	23477	4632	1429773	20078	GSM5251339	SRR14265443
Lvr_M_006_1	lane2_Lvr_M_6_1_1	35	2	9.4	Liver	Li	Lvr	M	6	1	Lvr-M-6	1260079	1602432	18831	891219	771	8704	27445	259666	33189	6165	1235028	14089	GSM5251340	SRR14265444
Lvr_M_006_2	lane2_Lvr_M_6_2_1	43	2.01	9.6	Liver	Li	Lvr	M	6	2	Lvr-M-6	1017506	1316723	16423	677591	598	7346	22236	241375	33290	5404	994037	13243	GSM5251341	SRR14265445
Lvr_M_006_3	lane4_Lvr_M_6_3_1	51	2.02	9.2	Liver	Li	Lvr	M	6	3	Lvr-M-6	983529	1261152	16691	672451	592	7378	19368	218495	28398	6787	958979	13369	GSM5251342	SRR14265446
Lvr_M_006_4	lane3_Lvr_M_6_4_1	59	2	9.8	Liver	Li	Lvr	M	6	4	Lvr-M-6	759929	993648	10724	546727	406	3468	13513	155811	16004	4778	743937	8498	GSM5251343	SRR14265447
Lvr_M_021_1	lane7_Lvr_M_21_1_1	37	1.95	9.3	Liver	Li	Lvr	M	21	1	Lvr-M-21	1231592	1407589	16674	895944	600	5509	18334	224370	27818	28546	1201808	13797	GSM5251344	SRR14265448
Lvr_M_021_2	lane8_Lvr_M_21_2_1	45	2.01	8.8	Liver	Li	Lvr	M	21	2	Lvr-M-21	993767	1303771	14768	687400	537	5568	19614	196788	27286	22813	959753	18993	GSM5251345	SRR14265449
Lvr_M_021_3	lane4_Lvr_M_21_3_1	53	2.02	8.5	Liver	Li	Lvr	M	21	3	Lvr-M-21	1244360	1501005	17304	931373	622	4396	18605	229141	21897	7499	1218679	13523	GSM5251346	SRR14265450
Lvr_M_021_4	lane7_Lvr_M_21_4_1	61	1.97	9.3	Liver	Li	Lvr	M	21	4	Lvr-M-21	1047336	1202342	14328	753645	515	4179	16780	195055	28014	21622	1020253	13198	GSM5251347	SRR14265451
Lvr_M_104_1	lane2_Lvr_M_100_1_1	39	2.04	9.1	Liver	Li	Lvr	M	104	1	Lvr-M-104	971394	1241655	20747	633690	694	8202	18327	237798	33389	7666	948653	10881	GSM5251348	SRR14265452
Lvr_M_104_2	lane3_Lvr_M_100_2_1	47	2.05	8.8	Liver	Li	Lvr	M	104	2	Lvr-M-104	1722802	2081980	28343	1213219	978	7976	24023	387820	31211	13879	1684446	15353	GSM5251349	SRR14265453
Lvr_M_104_3	lane1_Lvr_M_100_3_1	55	2.02	9.3	Liver	Li	Lvr	M	104	3	Lvr-M-104	1840947	2096596	28071	1243448	1026	15071	41540	408947	58955	8334	1776557	35555	GSM5251350	SRR14265454
Lvr_M_104_4	lane5_Lvr_M_100_4_1	63	2.02	9.5	Liver	Li	Lvr	M	104	4	Lvr-M-104	1508514	1678489	24688	1047564	897	10191	23894	326208	51805	9688	1479251	13579	GSM5251351	SRR14265455
Msc_F_002_1	lane1_Msc_F_2_1_1	98	2.03	9.4	Muscle	Mu	Msc	F	2	1	Msc-F-2	4390396	4926654	72039	3231997	1624	112989	61768	683759	88617	73180	4249988	64423	GSM5251352	SRR14265456
Msc_F_002_2	lane3_Msc_F_2_2_1	106	1.96	9.3	Muscle	Mu	Msc	F	2	2	Msc-F-2	3837067	4435018	45675	3001103	1202	36516	34181	611691	49692	28278	3750668	28729	GSM5251353	SRR14265457
Msc_F_002_3	lane7_Msc_F_2_3_1	114	1.96	9.3	Muscle	Mu	Msc	F	2	3	Msc-F-2	2290484	2584017	28413	1749760	846	22538	22089	376130	52957	19283	2235512	18468	GSM5251354	SRR14265458
Msc_F_002_4	lane1_Msc_F_2_4_1	122	1.97	9.4	Muscle	Mu	Msc	F	2	4	Msc-F-2	4369980	5038405	45857	3282327	1351	83332	56283	705184	88075	41114	4227727	66457	GSM5251355	SRR14265459
Msc_F_006_1	lane2_Msc_F_6_1_1	100	1.97	9.3	Muscle	Mu	Msc	F	6	1	Msc-F-6	2569282	3206245	42237	1774924	1087	20775	33149	509746	75607	89940	2510802	21817	GSM5251356	SRR14265460
Msc_F_006_2	lane1_Brn_F_6_2_1	76	1.98	9.6	Muscle	Mu	Msc	F	6	2	Msc-F-6	2970925	3435069	44207	2176436	1034	18083	42511	476400	59956	103392	2868831	48906	GSM5251357	SRR14265461
Msc_F_006_3	lane1_Msc_F_6_3_1	116	2	9	Muscle	Mu	Msc	F	6	3	Msc-F-6	2781802	3167831	51810	1976389	1372	15682	46571	481939	97191	65297	2691769	45551	GSM5251358	SRR14265462
Msc_F_006_4	lane5_Msc_F_6_4_1	124	1.99	9	Muscle	Mu	Msc	F	6	4	Msc-F-6	1823863	2008063	26138	1381304	677	14780	23235	281030	47368	32527	1784391	16804	GSM5251359	SRR14265463
Msc_F_021_1	lane7_Msc_F_21_1_1	101	1.99	8.6	Muscle	Mu	Msc	F	21	1	Msc-F-21	2504866	2859368	28912	1948295	840	6192	33370	391462	54993	16315	2459203	24487	GSM5251360	SRR14265464
Msc_F_021_2	lane6_Msc_F_21_2_1	110	2.07	9.1	Muscle	Mu	Msc	F	21	2	Msc-F-21	2334006	2540809	25270	1933557	684	4178	26906	267345	35138	15434	2290143	25494	GSM5251361	SRR14265465
Msc_F_021_3	lane2_Msc_F_21_3_1	118	2.03	8.9	Muscle	Mu	Msc	F	21	3	Msc-F-21	1803953	2147336	23819	1394630	657	4564	22401	271680	43795	25159	1772526	17248	GSM5251362	SRR14265466
Msc_F_021_4	lane5_Msc_F_21_4_1	126	2.01	9.1	Muscle	Mu	Msc	F	21	4	Msc-F-21	2921963	3195597	28658	2433292	767	6587	29284	326900	36393	39241	2881947	20841	GSM5251363	SRR14265467
Msc_F_104_1	lane4_Msc_F_100_1_1	104	1.98	8.8	Muscle	Mu	Msc	F	104	1	Msc-F-104	3446176	3945673	30844	2845123	997	9412	42549	404846	48681	31014	3386303	32710	GSM5251364	SRR14265468
Msc_F_104_2	lane3_Msc_F_100_2_1	112	2.05	9	Muscle	Mu	Msc	F	104	2	Msc-F-104	1891298	2141534	15931	1586169	437	2768	16406	203567	23081	27316	1859584	15623	GSM5251365	SRR14265469
Msc_F_104_3	lane6_Msc_F_100_3_1	120	2.05	8.8	Muscle	Mu	Msc	F	104	3	Msc-F-104	4531972	4977181	35474	3762126	1015	7696	49298	472468	38535	114773	4422819	50587	GSM5251366	SRR14265470
Msc_F_104_4	lane1_Msc_F_100_4_1	128	2.02	8.8	Muscle	Mu	Msc	F	104	4	Msc-F-104	3563637	4084514	36433	2804042	1063	9104	60365	456622	57514	75171	3458506	63323	GSM5251367	SRR14265471
Msc_M_002_1	lane8_Msc_M_2_1_1	97	1.87	9.6	Muscle	Mu	Msc	M	2	1	Msc-M-2	4624299	5328399	49677	3576757	1359	155328	54417	584009	71599	75318	4477572	55835	GSM5251368	SRR14265472
Msc_M_002_2	lane8_Msc_M_2_2_1	105	2.04	9.9	Muscle	Mu	Msc	M	2	2	Msc-M-2	3638106	4254467	38209	2784484	1207	116299	41185	513206	58176	39859	3521770	45481	GSM5251369	SRR14265473
Msc_M_002_3	lane2_Msc_M_2_3_1	113	1.91	9.3	Muscle	Mu	Msc	M	2	3	Msc-M-2	4435873	5422211	53802	2891792	1822	117903	61474	1071008	143034	54217	4327528	40821	GSM5251370	SRR14265474
Msc_M_002_4	lane2_Msc_M_2_4_1	121	1.96	9.1	Muscle	Mu	Msc	M	2	4	Msc-M-2	3937609	5103824	101246	2425816	2485	96025	218890	862081	73062	110452	3829084	47552	GSM5251371	SRR14265475
Msc_M_006_1	lane6_Msc_M_6_1_1	99	1.96	8.9	Muscle	Mu	Msc	M	6	1	Msc-M-6	3832507	4236038	56890	2780614	1394	17155	45088	671122	68530	150164	3701637	41550	GSM5251372	SRR14265476
Msc_M_006_2	lane7_Msc_M_6_2_1	107	1.94	9.2	Muscle	Mu	Msc	M	6	2	Msc-M-6	2958647	3230885	46121	2191832	1499	13494	49645	540298	58972	28855	2895473	27931	GSM5251373	SRR14265477
Msc_M_006_3	lane6_Msc_M_6_3_1	115	1.97	8.7	Muscle	Mu	Msc	M	6	3	Msc-M-6	3105364	3459211	44568	2307377	1267	11645	46350	564125	62065	34260	3030975	33707	GSM5251374	SRR14265478
Msc_M_006_4	lane7_Msc_M_6_4_1	123	1.95	9.1	Muscle	Mu	Msc	M	6	4	Msc-M-6	2134568	2380503	32191	1638030	806	10905	27153	339386	40398	28213	2091146	17486	GSM5251375	SRR14265479
Msc_M_021_1	lane5_Msc_M_21_1_1	102	2.01	7.6	Muscle	Mu	Msc	M	21	1	Msc-M-21	3906794	4249597	49894	3077130	1417	9129	48524	567912	84897	38320	3850234	29571	GSM5251376	SRR14265480
Msc_M_021_2	lane4_Msc_M_21_2_1	109	2.09	8.7	Muscle	Mu	Msc	M	21	2	Msc-M-21	1925954	2223628	22335	1546232	599	3617	24643	271545	29275	7589	1889726	20119	GSM5251377	SRR14265481
Msc_M_021_3	lane5_Msc_M_21_3_1	117	2.09	9.6	Muscle	Mu	Msc	M	21	3	Msc-M-21	1099844	1209643	10951	913347	304	2437	13013	122280	22938	3052	1080333	11522	GSM5251378	SRR14265482
Msc_M_021_4	lane4_Msc_M_21_4_1	125	2.03	8.9	Muscle	Mu	Msc	M	21	4	Msc-M-21	4007658	4442708	46891	3161129	1208	9686	52753	540275	60267	89321	3917691	46128	GSM5251379	SRR14265483
Msc_M_104_1	lane4_Msc_M_100_1_1	103	1.98	8.9	Muscle	Mu	Msc	M	104	1	Msc-M-104	4318310	4761888	48662	3355330	1376	9870	56811	602745	63975	133313	4218411	46228	GSM5251380	SRR14265484
Msc_M_104_2	lane3_Msc_M_100_2_1	111	2	8.7	Muscle	Mu	Msc	M	104	2	Msc-M-104	2268682	2745012	28824	1756290	860	4143	25116	371852	44382	15750	2230520	21465	GSM5251381	SRR14265485
Msc_M_104_3	lane3_Msc_M_100_3_1	119	2.03	8.6	Muscle	Mu	Msc	M	104	3	Msc-M-104	3965378	4379535	39771	2645389	1203	8567	51791	876069	44291	255762	3795775	42535	GSM5251382	SRR14265486
Msc_M_104_4	lane8_Msc_M_100_4_1	127	2.05	9.2	Muscle	Mu	Msc	M	104	4	Msc-M-104	4225480	4773087	40351	3457357	1152	6743	49412	497788	40676	83237	4135635	48764	GSM5251383	SRR14265487
Spl_F_002_1	lane3_Spl_F_2_1_1	2	2.05	3.7	Spleen	Sp	Spl	F	2	1	Spl-F-2	2706404	3351073	49047	1807761	2209	17377	54717	679030	51808	26137	2657165	18318	GSM5251384	SRR14265488
Spl_F_002_2	lane7_Spl_F_2_2_1	10	2.06	3.6	Spleen	Sp	Spl	F	2	2	Spl-F-2	2280728	2656928	34831	1578584	1620	16181	49582	486005	46720	50982	2235025	16223	GSM5251385	SRR14265489
Spl_F_002_3	lane4_Spl_F_2_3_1	18	2.04	2.4	Spleen	Sp	Spl	F	2	3	Spl-F-2	3213303	3970419	50875	2235291	2265	16793	63412	756081	45593	18590	3154446	24403	GSM5251386	SRR14265490
Spl_F_002_4	lane8_Spl_F_2_4_1	26	2.06	2.6	Spleen	Sp	Spl	F	2	4	Spl-F-2	3534614	4103416	42525	2545702	1893	20366	66054	722350	53775	45306	3455365	36643	GSM5251387	SRR14265491
Spl_F_006_1	lane8_Spl_F_6_1_1	4	2.05	9.1	Spleen	Sp	Spl	F	6	1	Spl-F-6	4099903	5096127	55063	3027416	2564	36255	83796	729970	82124	46824	4016703	35891	GSM5251388	SRR14265492
Spl_F_006_2	lane1_Spl_F_6_2_1	12	2.03	9.5	Spleen	Sp	Spl	F	6	2	Spl-F-6	1972880	2347560	30776	1444317	1518	19257	49366	346115	43661	12113	1924565	25757	GSM5251389	SRR14265493
Spl_F_006_3	lane7_Spl_F_6_3_1	20	2.05	8.9	Spleen	Sp	Spl	F	6	3	Spl-F-6	2016997	2287635	29166	1447999	1240	14859	40431	405457	38192	24111	1979806	15542	GSM5251390	SRR14265494
Spl_F_006_4	lane2_Spl_F_6_4_1	28	2.07	9.1	Spleen	Sp	Spl	F	6	4	Spl-F-6	2021121	2473388	32328	1404392	1537	19276	41640	451975	44753	13862	1993861	11358	GSM5251391	SRR14265495
Spl_F_021_1	lane8_Spl_F_21_1_1	6	2.03	7.8	Spleen	Sp	Spl	F	21	1	Spl-F-21	2760791	3479773	30890	1850701	1537	26242	53343	726781	37223	9399	2711663	24675	GSM5251392	SRR14265496
Spl_F_021_2	lane5_Spl_F_21_2_1	14	2.04	9	Spleen	Sp	Spl	F	21	2	Spl-F-21	3377013	3765947	31067	1805115	1513	45955	77435	1295566	59406	39403	3329662	21553	GSM5251393	SRR14265497
Spl_F_021_3	lane2_Spl_F_21_3_1	22	2.03	9.4	Spleen	Sp	Spl	F	21	3	Spl-F-21	2823704	3482627	29418	1689892	1460	37018	67059	912286	47373	23235	2789236	15963	GSM5251394	SRR14265498
Spl_F_021_4	lane7_Spl_F_21_4_1	30	2.03	7.8	Spleen	Sp	Spl	F	21	4	Spl-F-21	2673666	3003777	29415	1885218	1348	27061	51058	596825	43121	19922	2629489	19698	GSM5251395	SRR14265499
Spl_F_104_1	lane6_Spl_F_100_1_1	8	2.05	10	Spleen	Sp	Spl	F	104	1	Spl-F-104	3756239	4177065	67579	2495785	3129	23390	100535	877056	101421	51727	3680904	35617	GSM5251396	SRR14265500
Spl_F_104_2	lane2_Spl_F_100_2_1	16	1.99	8.7	Spleen	Sp	Spl	F	104	2	Spl-F-104	4498497	5727520	36785	1482510	1761	34239	78605	2712445	104704	19922	4447196	27526	GSM5251397	SRR14265501
Spl_F_104_3	lane2_Spl_F_100_3_1	24	2.01	7.5	Spleen	Sp	Spl	F	104	3	Spl-F-104	3565149	4631831	35913	1506271	1612	28442	89335	1776143	95759	9888	3522843	21786	GSM5251398	SRR14265502
Spl_F_104_4	lane3_Spl_F_100_4_1	32	2.03	8.1	Spleen	Sp	Spl	F	104	4	Spl-F-104	14682338	17138744	166997	8601224	9559	139366	468439	4908618	182203	90292	14433835	115640	GSM5251399	SRR14265503
Spl_M_002_1	lane5_Spl_M_2_1_1	1	2.04	2.2	Spleen	Sp	Spl	M	2	1	Spl-M-2	2900950	3514582	32269	1713994	1445	15556	45558	916096	125958	27448	2851889	22626	GSM5251400	SRR14265504
Spl_M_002_2	lane1_Spl_M_2_2_1	9	2.04	5.1	Spleen	Sp	Spl	M	2	2	Spl-M-2	2723297	3358962	26873	1921417	1312	14850	48378	565675	53196	54128	2644579	37468	GSM5251401	SRR14265505
Spl_M_002_3	lane6_Spl_M_2_3_1	17	2.04	2.6	Spleen	Sp	Spl	M	2	3	Spl-M-2	2950967	3730094	30785	1787246	1397	10693	43986	916354	101568	29615	2887657	29323	GSM5251402	SRR14265506
Spl_M_002_4	lane5_Spl_M_2_4_1	25	2.04	2.4	Spleen	Sp	Spl	M	2	4	Spl-M-2	1862990	2181059	22918	1147116	994	10811	33321	510205	93800	28086	1827372	15739	GSM5251403	SRR14265507
Spl_M_006_1	lane1_Spl_M_6_1_1	3	2.02	9.5	Spleen	Sp	Spl	M	6	1	Spl-M-6	3058921	3524526	43439	2182654	1991	25502	78012	584792	70637	30827	2982837	41067	GSM5251404	SRR14265508
Spl_M_006_2	lane4_Spl_M_6_2_1	11	2.03	9.5	Spleen	Sp	Spl	M	6	2	Spl-M-6	3552554	4064021	50029	2722979	2388	24601	69391	578741	51431	23270	3487232	29724	GSM5251405	SRR14265509
Spl_M_006_3	lane8_Spl_M_6_3_1	19	2.06	9.6	Spleen	Sp	Spl	M	6	3	Spl-M-6	2807285	3377734	34696	2133649	1634	24073	55551	436688	53056	43460	2750187	24478	GSM5251406	SRR14265510
Spl_M_006_4	lane5_Spl_M_6_4_1	27	2.05	9	Spleen	Sp	Spl	M	6	4	Spl-M-6	2242707	2442713	28590	1674003	1325	17799	43257	369237	37281	58200	2205690	13015	GSM5251407	SRR14265511
Spl_M_021_1	lane3_Spl_M_21_1_1	5	2.05	8.5	Spleen	Sp	Spl	M	21	1	Spl-M-21	1830671	2126299	22652	1160347	1086	24867	39148	526944	30165	13130	1801527	12332	GSM5251408	SRR14265512
Spl_M_021_2	lane4_Spl_M_21_2_1	13	2.04	9.9	Spleen	Sp	Spl	M	21	2	Spl-M-21	3715796	4211436	46108	2253404	2206	47044	79715	1166180	67660	29598	3658373	23881	GSM5251409	SRR14265513
Spl_M_021_3	lane1_Spl_M_21_3_1	21	2.04	9.4	Spleen	Sp	Spl	M	21	3	Spl-M-21	2968725	3379822	30015	1745952	1386	36145	77652	938084	56634	43632	2894219	39225	GSM5251410	SRR14265514
Spl_M_021_4	lane6_Spl_M_21_4_1	29	2.04	8.6	Spleen	Sp	Spl	M	21	4	Spl-M-21	3661066	4089843	38813	2482831	1887	27776	62560	962587	42915	15720	3604880	25977	GSM5251411	SRR14265515
Spl_M_104_1	lane3_Spl_M_100_1_1	7	2.05	9.3	Spleen	Sp	Spl	M	104	1	Spl-M-104	2610138	3166504	28719	1568631	1425	20464	46335	892992	29808	7389	2577327	14375	GSM5251412	SRR14265516
Spl_M_104_2	lane6_Spl_M_100_2_1	15	2.04	9.8	Spleen	Sp	Spl	M	104	2	Spl-M-104	3210138	3649831	33638	2396574	1561	21747	70103	576413	67423	16247	3153429	26432	GSM5251413	SRR14265517
Spl_M_104_3	lane4_Spl_M_100_3_1	23	2.03	8.8	Spleen	Sp	Spl	M	104	3	Spl-M-104	2152214	2620986	29778	1323355	1522	23288	48215	661041	41175	9466	2120766	14374	GSM5251414	SRR14265518
Spl_M_104_4	lane7_Spl_M_100_4_1	31	NA	9.3	Spleen	Sp	Spl	M	104	4	Spl-M-104	2459279	2804185	20385	1326558	898	20077	54437	958095	39516	22875	2421934	16438	GSM5251415	SRR14265519
Thm_F_002_1	lane5_Thm_F_2_1_1	258	2.06	10	Thymus	Th	Thm	F	2	1	Thm-F-2	3305816	3674040	59031	2445671	2823	22415	64758	572660	112354	10523	3266798	15581	GSM5251416	SRR14265520
Thm_F_002_2	lane6_Thm_F_2_2_1	266	2.05	10	Thymus	Th	Thm	F	2	2	Thm-F-2	3600257	3951841	55394	2726079	2745	17173	61091	611419	90243	11166	3538364	24947	GSM5251417	SRR14265521
Thm_F_002_3	lane7_Thm_F_2_3_1	274	2.05	10	Thymus	Th	Thm	F	2	3	Thm-F-2	2440617	2829855	38956	1847937	2007	15427	45207	405215	63730	7575	2403748	14563	GSM5251418	SRR14265522
Thm_F_002_4	lane4_Thm_F_2_4_1	282	2.06	10	Thymus	Th	Thm	F	2	4	Thm-F-2	2407857	2836062	33980	1881889	1713	14376	39107	359074	52063	9076	2367222	16579	GSM5251419	SRR14265523
Thm_F_006_1	lane3_Thm_F_6_1_1	260	2.05	10	Thymus	Th	Thm	F	6	1	Thm-F-6	2749078	3139946	42659	2201018	2178	14539	41118	381625	46306	9202	2718432	10433	GSM5251420	SRR14265524
Thm_F_006_2	lane1_Thm_F_6_2_1	268	2.05	10	Thymus	Th	Thm	F	6	2	Thm-F-6	2478554	2915932	41965	1929897	2183	14964	51356	355447	43571	11593	2422253	27578	GSM5251421	SRR14265525
Thm_F_006_3	lane4_Thm_F_6_3_1	276	2.06	10	Thymus	Th	Thm	F	6	3	Thm-F-6	3208181	3608557	53644	2401740	2706	19180	74817	550250	66206	16147	3158408	23491	GSM5251422	SRR14265526
Thm_F_006_4	lane2_Thm_F_6_4_1	284	2.06	9.3	Thymus	Th	Thm	F	6	4	Thm-F-6	2872328	3640970	56218	1972106	2831	23124	64381	643189	79621	12992	2832956	17866	GSM5251423	SRR14265527
Thm_F_021_1	lane5_Thm_F_21_1_1	262	2.06	10	Thymus	Th	Thm	F	21	1	Thm-F-21	3147589	3484478	46179	2437476	2289	17137	52607	505078	59562	11612	3112076	15649	GSM5251424	SRR14265528
Thm_F_021_2	lane7_Thm_F_21_2_1	270	2.05	10	Thymus	Th	Thm	F	21	2	Thm-F-21	2615908	2933078	41930	2055646	2013	14871	45156	380122	46436	13946	2578693	15788	GSM5251425	SRR14265529
Thm_F_021_3	lane7_Thm_F_21_3_1	278	2.05	10	Thymus	Th	Thm	F	21	3	Thm-F-21	3440091	3754521	46285	2783674	2342	17109	51857	456245	55820	8021	3395253	18738	GSM5251426	SRR14265530
Thm_F_021_4	lane1_Thm_F_21_4_1	286	2.06	9.8	Thymus	Th	Thm	F	21	4	Thm-F-21	3067046	3571577	46393	2377747	2327	18287	61369	443639	68411	14214	3000313	34659	GSM5251427	SRR14265531
Thm_F_104_1	lane2_Thm_F_100_1_1	264	2.05	9.2	Thymus	Th	Thm	F	104	1	Thm-F-104	3039698	4021322	46835	1795005	2142	18114	57577	890304	119712	78549	2953666	31460	GSM5251428	SRR14265532
Thm_F_104_2	lane3_Thm_F_100_2_1	272	2.02	9.7	Thymus	Th	Thm	F	104	2	Thm-F-104	3005510	3698262	48729	2028584	2095	14850	54997	725968	58862	48814	2945273	22611	GSM5251429	SRR14265533
Thm_F_104_3	lane6_Thm_F_100_3_1	280	2	9.4	Thymus	Th	Thm	F	104	3	Thm-F-104	3722355	4161057	55223	2764919	2512	17379	62481	679876	68853	40120	3658883	30992	GSM5251430	SRR14265534
Thm_F_104_4	lane6_Thm_F_100_4_1	288	2.03	9.2	Thymus	Th	Thm	F	104	4	Thm-F-104	3631362	4276133	60736	2348406	2956	21358	74049	938706	122067	26607	3559843	36477	GSM5251431	SRR14265535
Thm_M_002_1	lane8_Thm_M_2_1_1	257	2.06	10	Thymus	Th	Thm	M	2	1	Thm-M-2	3019805	3744541	39236	2428918	2045	15903	46594	405330	46673	13504	2965147	21602	GSM5251432	SRR14265536
Thm_M_002_2	lane2_Thm_M_2_2_1	265	2.06	10	Thymus	Th	Thm	M	2	2	Thm-M-2	2172326	2662251	31963	1667586	1743	20235	39549	346359	42804	9519	2140030	12568	GSM5251433	SRR14265537
Thm_M_002_3	lane1_Thm_M_2_3_1	273	2.04	10	Thymus	Th	Thm	M	2	3	Thm-M-2	2725588	3154906	35173	2108818	1926	18527	54419	396274	59748	16281	2656530	34422	GSM5251434	SRR14265538
Thm_M_002_4	lane5_Thm_M_2_4_1	281	2.06	10	Thymus	Th	Thm	M	2	4	Thm-M-2	1887734	2104725	23094	1534504	1191	11113	29028	229216	37945	10080	1859906	11563	GSM5251435	SRR14265539
Thm_M_006_1	lane7_Thm_M_6_1_1	259	2.06	10	Thymus	Th	Thm	M	6	1	Thm-M-6	2706563	3065064	43262	2055941	2106	15035	48121	452745	63705	10265	2668886	15383	GSM5251436	SRR14265540
Thm_M_006_2	lane8_Thm_M_6_2_1	267	2.06	9.8	Thymus	Th	Thm	M	6	2	Thm-M-6	3555666	4043414	59668	2751271	2961	20628	68812	544206	66310	10805	3485244	31005	GSM5251437	SRR14265541
Thm_M_006_3	lane1_Thm_M_6_3_1	275	2.06	9.8	Thymus	Th	Thm	M	6	3	Thm-M-6	3085429	3449342	49047	2351397	2666	21619	93556	440116	76819	12458	3012756	37751	GSM5251438	SRR14265542
Thm_M_006_4	lane6_Thm_M_6_4_1	283	2.06	9.7	Thymus	Th	Thm	M	6	4	Thm-M-6	1194504	1386797	22686	878460	1166	7359	28168	213179	17805	16356	1170897	9325	GSM5251439	SRR14265543
Thm_M_021_1	lane2_Thm_M_21_1_1	261	2.06	10	Thymus	Th	Thm	M	21	1	Thm-M-21	2584307	3054720	40517	2064900	2057	16377	41883	344085	49530	13690	2556390	11268	GSM5251440	SRR14265544
Thm_M_021_2	lane3_Thm_M_21_2_1	269	2.05	10	Thymus	Th	Thm	M	21	2	Thm-M-21	2016113	2287760	32021	1562306	1535	12988	36654	311510	39448	7700	1989274	11951	GSM5251441	SRR14265545
Thm_M_021_3	lane8_Thm_M_21_3_1	277	2.04	9.9	Thymus	Th	Thm	M	21	3	Thm-M-21	2914456	3464788	40149	2306020	2078	16742	53461	398816	57775	14114	2861334	25301	GSM5251442	SRR14265546
Thm_M_021_4	lane4_Thm_M_21_4_1	285	2.06	9.9	Thymus	Th	Thm	M	21	4	Thm-M-21	3446840	3975628	50349	2727895	2512	14363	55184	510469	43824	19347	3396210	22897	GSM5251443	SRR14265547
Thm_M_104_1	lane8_Thm_M_100_1_1	263	2.04	9.3	Thymus	Th	Thm	M	104	1	Thm-M-104	2735164	3633845	35326	1939430	1576	12440	56283	555733	80361	14993	2671872	39022	GSM5251444	SRR14265548
Thm_M_104_2	lane3_Thm_M_100_2_1	271	2.07	9.4	Thymus	Th	Thm	M	104	2	Thm-M-104	2235099	2860042	40978	1443476	1984	10955	38963	613409	53772	14827	2201620	16735	GSM5251445	SRR14265549
Thm_M_104_3	lane5_Thm_M_100_3_1	279	2.01	9.5	Thymus	Th	Thm	M	104	3	Thm-M-104	2838993	3079880	34328	2075514	1553	13285	73562	513615	58488	48162	2780712	20486	GSM5251446	SRR14265550
Thm_M_104_4	lane4_Thm_M_100_4_1	287	2.05	9.1	Thymus	Th	Thm	M	104	4	Thm-M-104	4328259	5002608	74208	2834489	3135	25939	91483	1054390	131270	68932	4240231	44413	GSM5251447	SRR14265551
Tst_M_002_1	lane4_Tst_M_2_1_1	225	2.04	10	Testes	Te	Tst	M	2	1	Tst-M-2	4561097	5092382	60141	3357734	2833	42216	80968	849728	97031	37656	4481566	32790	GSM5251448	SRR14265552
Tst_M_002_2	lane8_Tst_M_2_2_1	233	2.05	10	Testes	Te	Tst	M	2	2	Tst-M-2	3377830	4091091	49007	2385232	2440	40790	69729	664373	91717	41009	3305748	33533	GSM5251449	SRR14265553
Tst_M_002_3	lane8_Tst_M_2_3_1	241	2.04	9.7	Testes	Te	Tst	M	2	3	Tst-M-2	4618364	5516158	53527	3394267	2731	38420	77003	896253	83948	32854	4527514	39361	GSM5251450	SRR14265554
Tst_M_002_4	lane6_Tst_M_2_4_1	249	2.05	10	Testes	Te	Tst	M	2	4	Tst-M-2	5143842	5767793	79222	3621580	3675	43826	104534	1115858	109378	25050	5050799	40719	GSM5251451	SRR14265555
Tst_M_006_1	lane2_Tst_M_6_1_1	227	2.08	9.6	Testes	Te	Tst	M	6	1	Tst-M-6	18120685	19297804	297858	1070286	34888	406869	2594554	13400081	35086	16610	17733347	264453	GSM5251452	SRR14265556
Tst_M_006_2	lane2_Tst_M_6_2_1	235	2.07	9.5	Testes	Te	Tst	M	6	2	Tst-M-6	18598759	19739391	302364	1115090	34768	417164	2641886	13764292	31696	9926	18192413	281573	GSM5251453	SRR14265557
Tst_M_006_3	lane5_Tst_M_6_3_1	243	2.05	9.5	Testes	Te	Tst	M	6	3	Tst-M-6	12901598	13463650	202032	734193	22824	279874	1791612	9642120	25242	13230	12618905	190471	GSM5251454	SRR14265558
Tst_M_006_4	lane4_Tst_M_6_4_1	251	2.06	9.3	Testes	Te	Tst	M	6	4	Tst-M-6	14211234	15148970	232467	1046076	25122	302904	1943621	10355964	28983	12587	13830645	263510	GSM5251455	SRR14265559
Tst_M_021_1	lane3_Tst_M_21_1_1	229	2.06	9.3	Testes	Te	Tst	M	21	1	Tst-M-21	16337514	17238582	260452	1091796	29612	338534	2149046	12191982	48414	8191	16000664	219487	GSM5251456	SRR14265560
Tst_M_021_2	lane2_Tst_M_21_2_1	237	2.06	9.3	Testes	Te	Tst	M	21	2	Tst-M-21	15523557	16742387	251644	1186440	27673	327469	2155852	11272039	62705	11951	15188916	227784	GSM5251457	SRR14265561
Tst_M_021_3	lane7_Tst_M_21_3_1	245	2.05	9	Testes	Te	Tst	M	21	3	Tst-M-21	16068006	16991593	256936	1244799	28463	319884	2032486	11845239	68248	13647	15682644	258304	GSM5251458	SRR14265562
Tst_M_021_4	lane7_Tst_M_21_4_1	253	2.06	9.4	Testes	Te	Tst	M	21	4	Tst-M-21	18378089	19423379	286620	1413546	32605	358364	2307496	13597766	79163	12917	17945620	289612	GSM5251459	SRR14265563
Tst_M_104_1	lane2_Tst_M_100_1_1	231	2.05	9.6	Testes	Te	Tst	M	104	1	Tst-M-104	6136291	6871571	37778	4464060	2609	54327	154195	1318835	62991	10064	6074888	31432	GSM5251460	SRR14265564
Tst_M_104_2	lane1_Tst_M_100_2_1	239	2.06	9.5	Testes	Te	Tst	M	104	2	Tst-M-104	6734680	7385609	33088	5633398	1740	45092	103503	771978	61603	13517	6608392	70761	GSM5251461	SRR14265565
Tst_M_104_3	lane7_Tst_M_100_3_1	247	2.06	9.6	Testes	Te	Tst	M	104	3	Tst-M-104	8025400	8518505	24419	7310154	1232	46221	49176	498209	32558	27366	7943039	36065	GSM5251462	SRR14265566
Tst_M_104_4	lane6_Tst_M_100_4_1	255	2.05	9.2	Testes	Te	Tst	M	104	4	Tst-M-104	8155212	8691415	26276	7383279	1237	36248	53132	572748	37328	4895	8067402	40069	GSM5251463	SRR14265567
Utr_F_002_1	lane1_Utr_F_2_1_1	226	1.97	9.1	Uterus	Ut	Utr	F	2	1	Utr-F-2	7351967	8566842	55013	5630099	2432	35087	88126	1213388	202905	37614	7195624	87303	GSM5251464	SRR14265568
Utr_F_002_2	lane4_Utr_F_2_2_1	234	1.89	9.1	Uterus	Ut	Utr	F	2	2	Utr-F-2	9092230	10208844	74386	6814562	3193	39611	93735	1696787	259406	44355	8953423	66195	GSM5251465	SRR14265569
Utr_F_002_3	lane3_Utr_F_2_3_1	242	2.02	9.1	Uterus	Ut	Utr	F	2	3	Utr-F-2	3259356	4113821	26365	2310018	1096	14183	28921	757005	78751	24754	3212601	18263	GSM5251466	SRR14265570
Utr_F_002_4	lane6_Utr_F_2_4_1	250	1.95	9.6	Uterus	Ut	Utr	F	2	4	Utr-F-2	4030535	4576941	42964	2865118	2028	18029	55949	846456	131214	33921	3952486	34856	GSM5251467	SRR14265571
Utr_F_006_1	lane3_Utr_F_6_1_1	228	1.98	9.6	Uterus	Ut	Utr	F	6	1	Utr-F-6	7493142	8298302	54175	6291236	2113	24356	61333	902624	92183	34079	7415432	31043	GSM5251468	SRR14265572
Utr_F_006_2	lane5_Utr_F_6_2_1	236	1.97	9.6	Uterus	Ut	Utr	F	6	2	Utr-F-6	6265079	6969299	67430	4717066	2663	23101	67249	1119134	193912	45407	6199357	29117	GSM5251469	SRR14265573
Utr_F_006_3	lane5_Utr_F_6_3_1	244	1.97	9.6	Uterus	Ut	Utr	F	6	3	Utr-F-6	6465822	6933674	37421	5551945	1640	17348	46044	626172	125398	35244	6405541	24610	GSM5251470	SRR14265574
Utr_F_006_4	lane1_Utr_F_6_4_1	252	2.02	9.7	Uterus	Ut	Utr	F	6	4	Utr-F-6	6156427	7116143	54587	4913719	2181	24184	78272	851538	150256	23980	6046202	57710	GSM5251471	SRR14265575
Utr_F_021_1	lane4_Utr_F_21_1_1	230	2.05	9.7	Uterus	Ut	Utr	F	21	1	Utr-F-21	7940066	8889915	72195	6589919	2729	20518	74846	1030111	73142	34051	7838592	42555	GSM5251472	SRR14265576
Utr_F_021_2	lane6_Utr_F_21_2_1	238	2.04	9.7	Uterus	Ut	Utr	F	21	2	Utr-F-21	8855251	9662116	51048	7686898	2133	24559	74501	825911	104715	38108	8741263	47378	GSM5251473	SRR14265577
Utr_F_021_3	lane3_Utr_F_21_3_1	246	1.9	9.8	Uterus	Ut	Utr	F	21	3	Utr-F-21	7547345	8507707	50975	6428893	2130	18466	57248	855831	88975	15816	7475288	29011	GSM5251474	SRR14265578
Utr_F_021_4	lane8_Utr_F_21_4_1	254	2.05	9.7	Uterus	Ut	Utr	F	21	4	Utr-F-21	8083820	9135660	52431	6967427	2175	30734	76672	751168	80503	68599	7959572	54111	GSM5251475	SRR14265579
Utr_F_104_1	lane5_Utr_F_100_1_1	232	1.88	9.7	Uterus	Ut	Utr	F	104	1	Utr-F-104	11675483	12390316	57872	10571771	2230	27648	78277	775093	97314	25669	11574668	39609	GSM5251476	SRR14265580
Utr_F_104_2	lane7_Utr_F_100_2_1	240	1.97	9.5	Uterus	Ut	Utr	F	104	2	Utr-F-104	7395670	8117464	49133	6444291	2009	25332	59118	690330	77844	18043	7319272	29570	GSM5251477	SRR14265581
Utr_F_104_3	lane8_Utr_F_100_3_1	248	1.95	9.6	Uterus	Ut	Utr	F	104	3	Utr-F-104	9521344	10347657	61601	8232079	2433	27463	90660	932505	85536	30172	9383426	58895	GSM5251478	SRR14265582
Utr_F_104_4	lane1_Utr_F_100_4_1	256	1.99	9.4	Uterus	Ut	Utr	F	104	4	Utr-F-104	9480790	10304503	61999	8179994	2550	32947	114937	869105	109712	24159	9315953	85387	GSM5251479	SRR14265583
